# eDNA and microscopy reveal traits underlying long-term zooplankton community change

**DOI:** 10.1038/s42003-026-10901-w

**Published:** 2026-10-27

**Authors:** Karen Tait, Penelope K. Lindeque, Amanda Beesley, Andrea McEvoy, Helen Parry, Angus Atkinson

**Affiliations:** Plymouth Marine Laboratory, Prospect Place, Plymouth, UK

**Keywords:** Biooceanography, Climate-change ecology, Molecular ecology

## Abstract

Zooplankton form a vital link between phytoplankton and higher trophic levels, including commercially exploited fish, making recent declines in copepods and increases in meroplankton a major concern. To investigate underlying mechanisms, we compared weekly, high-resolution net (microscope analysis: 35 years) and surface water (eDNA: 11 years) from Station L4 in the Western English Channel. The two methods showed strong agreement for holoplanktonic crustaceans and gelatinous predators, but weaker correspondence for meroplankton. The far greater taxonomic insight from meroplankton groups provided by eDNA revealed a shift towards higher relative sequence abundance of soft sediment (mainly sublittoral) fauna, rather than from rocky nearshore. Trait-based analysis showed long-term increases were mainly fine filter feeders, including many meroplankton larvae. This suggests that changes in water column processes, linked to declining nutrient supply and a shift towards smaller phytoplankton, were contributing to broader changes in the pelagic food web and benthic-pelagic coupling.

## Introduction

Zooplankton constitute 40% of total marine animal biomass, are the main link between phytoplankton and higher trophic levels including commercially exploited fish and may account for 85% of carbon sinking to the deep ocean^1^. However, climatic warming is already greatly impacting zooplankton^2^, and projected declines can amplify into relatively larger declines in their consumers, such as fish, marine mammals and birds^3^. Both models^4^ and observations^5,6^ show frequent declines in key crustacean groups such as copepods and euphausiids (krill), often with partial replacement by more carnivorous or gelatinous forms^7^ or meroplankton, the pelagic larvae of benthic invertebrates^2,8^. A variety of contrasting mechanisms have been proposed. These include direct effects of warming on distributions^9,10^, direct temperature effects on species interactions and bentho-pelagic coupling^8^ and nutrient-mediated effects on food web size- or taxonomic structure^7,11^. While we clearly need a better mechanistic understanding of these profound readjustments, a key impediment has been a lack of highly resolved zooplankton functional trait data. Coarse taxonomic- and temporal resolution, particularly of the meroplankton, has limited our ability to identify the common traits among taxa showing contrasting trends under climatic change, as larvae are often identified to broad taxonomic levels by microscopy.

To improve our resolution of plankton taxonomy and traits, the use of environmental DNA (eDNA) has rapidly been gathering pace with many researchers now applying eDNA for the rapid assessment of whole communities^12^. eDNA techniques can produce valuable data on groups that are hard to identify without specialist taxonomic knowledge, are difficult to sample, are rare or are previously unstudied^13^. In addition to the scientific advantages, shifts away from the net sampling and microscopic analysis are being driven by declining funding for traditional methods, and improved funding for newer methods perceived by some as lower cost and not requiring dedicated research cruises^14,15^. However, fluctuating rates of DNA degradation, transportation of DNA by water currents, methodological variability and a lack of standardised protocols^16–18^ mean that eDNA-derived data provide a different perspective on plankton community structure rather than a direct replacement for microscope-based analyses. As a result, proof of concept studies that directly compare data derived from microscope and molecular methods are essential for understanding how these approaches can be combined to monitor and assess ecosystem health.

The station L4 off Plymouth has been sampled on a weekly basis since 1988, with a full suite of physical, chemical and biological variables including taxonomic identification of ~400 taxa^19^. This 35-year time series has shown profound changes that are mirrored more widely across the NE Atlantic, including declines in the classic food web components such as diatoms and copepods, and increases in meroplankton^2,11^. Since 2012, a parallel eDNA data set has been obtained by surface water sampling, also on a weekly resolution, allowing direct comparison with zooplankton with our microscopically identified full-depth net samples, based on 415 concurrently collected samples over an 11-year period. Both nets and eDNA have major strengths and limitations. Importantly, our eDNA- and microscope-based methods sample different components of the water column. They also quantify fundamentally different properties of plankton communities (e.g. sequence vs numerical abundance), such that exact correspondence between methods is not expected. Our objective was to combine these approaches for a more holistic view of the structure, seasonality and trends in the plankton, to gain a better taxonomic appreciation of species that are increasing or decreasing. Agreement between the two approaches would provide strong evidence of shared temporal patterns. Assigning a feeding mode to zooplankton taxa provides a framework for investigating changes in trophic structure within pelagic ecosystems. The integration of eDNA- and microscope-derived data allows zooplankton dynamics to be viewed from different methodological perspectives, as the two methods differ in their sensitivity to taxonomic groups and life-stages. Used together, they provide an opportunity to better characterise patterns of change within complex plankton communities.

## Results

### Finer zooplankton taxonomic resolution can be detected using eDNA with temporal trends in agreement between molecular and morphological methods

**Fig. 1:**
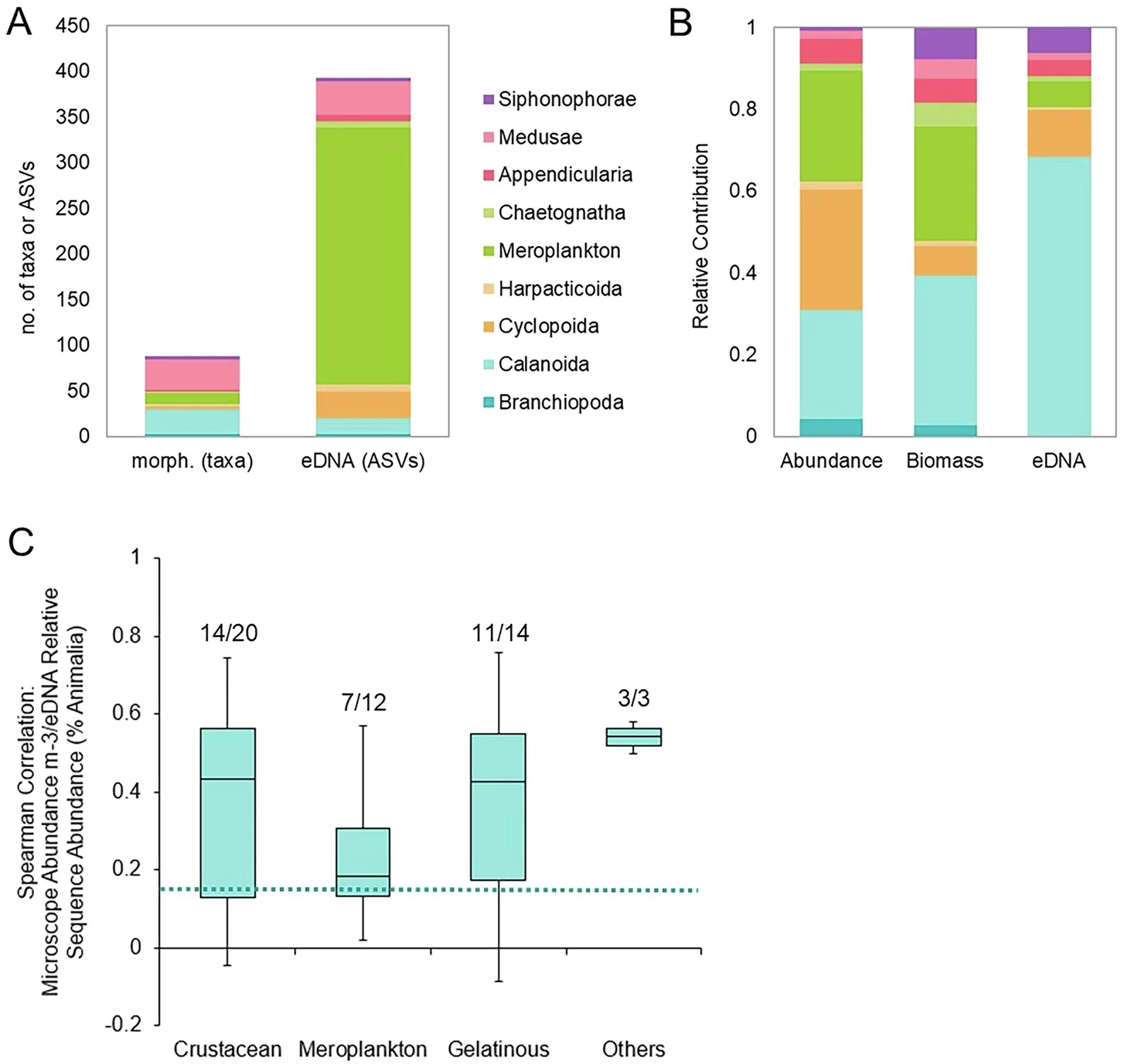
Comparison of zooplankton data from microscope and molecular methods. **A** Number of taxonomic units identified by microscopy and eDNA (*n* = 415). **B** Zooplankton composition expressed as relative sequence abundance (eDNA) and relative abundance and biomass (microscopy) (*n* = 415). **C** Correspondence between eDNA- and morphology-derived monthly occurrence patterns, assessed using Spearman rank correlation analysis of monthly averaged timeseries (*n* = 125). Taxa were grouped as Crustaceans, Meroplankton, Gelatinous Zooplankton, Others (see Methods for definitions). Boxplots show the distribution of correlation coefficients within each group; the central line indicates the median, box boundaries represent the 25th and 75th percentiles, and whiskers indicate the minimum and maximum values. Numbers above each box indicate the number of taxa exhibiting significant correlations relative to the total number of taxa analysed in that group.Numbers above each bar indicate the total number of taxa in each group and the subset exhibiting significant correlations. The blue dashed line indicates the Spearman correlation coefficient threshold corresponding to statistical significance (*p* = 0.05).

To provide additional taxonomic insight into drivers of zooplankton community change, weekly eDNA samples spanning February 2012 and March 2023 were analysed in detail. Our 18S rRNA V9 sequence data spanned 15 Phyla (Supplementary Data 1). The majority (81%) of Animalia eDNA sequences were identified as holoplankton crustacean taxa, grouping to 61 (14.3%) of the 427 Animalia Amplicon Sequence Variants (ASVs) identified (Fig. 1). Many could be identified to Genus level (Supplementary Data 1). The higher representation of Calanoida sequences makes it difficult to compare relative contributions of other taxa, and so taxa were ranked according to their abundance (Supplementary Table 1). This highlights the much lower proportional representation of Branchiopoda, Cirripedia and Decapoda within the molecular dataset, and the higher proportional representation of meroplankton taxa Ascidiacea, Anthozoa, Bivalvia and Ctenophora (Supplementary Table 1).

eDNA identified 55 Cnidaria ASVs (12.6% of Animalia ASVs) (Fig. 1; Supplementary Tables 1 and 2). Assigning taxonomy to Genus level for some Cnidaria ASVs was not possible, with several mismatches between the two datasets. However, more confident matches could be made for some taxa present in higher abundance (e.g. *Lizzia* sp., *Obelia* sp., *Liriope* sp., *Solmaris* sp., *Aglantha* sp. and *Muggiaea* sp.; Supplementary Data 1). For Appendicularia, Chaetognatha and Ctenophora, not all individuals can be classified by microscopy. eDNA was able to match the taxa that can be confidently identified by microscopy and provided further insight into those individuals that cannot be identified by microscopy (Supplementary Tables 1 and 2). A notable exception was the absence of Salpida within the eDNA data. DNA gene V9 Salpida DNA sequences are present within NCBI nucleotide databases, but no eDNA for Salpida could be detected, likely reflecting their patchy distribution, brief occurrence at station L4 during the summer of 2022 only, and limited representation in surface-water samples.

Meroplankton are identified to broad taxonomic levels, often only to Phylum level, by microscopy. Here, eDNA provides enhanced taxonomic resolution that is not captured within the L4 microscope data. The majority of ASVs were identified as meroplankton (277 ASVs; 62%) (Fig. 1a; Supplementary Tables 1 and 2). This includes Polychaeta, Sipuncula, Decapoda, Cirripedia, Ascidiacea, Anthozoa, Teleostei, Echinodermata, Bivalvia, Gastropoda, Nemertea, Phoronida and Platyhelminths. These ASVs could be identified to Class, Order or even Family level depending on the taxonomic grouping (Supplementary Table 1). Of note was the high number of Polychaeta ASVs identified: 100 ASVs (23.7%) of all Animalia ASVs (Supplementary Table 1). A small number of Porifera eDNA sequences were detected; Porifera do not feature in the morphological time-series.

**Fig. 2:**
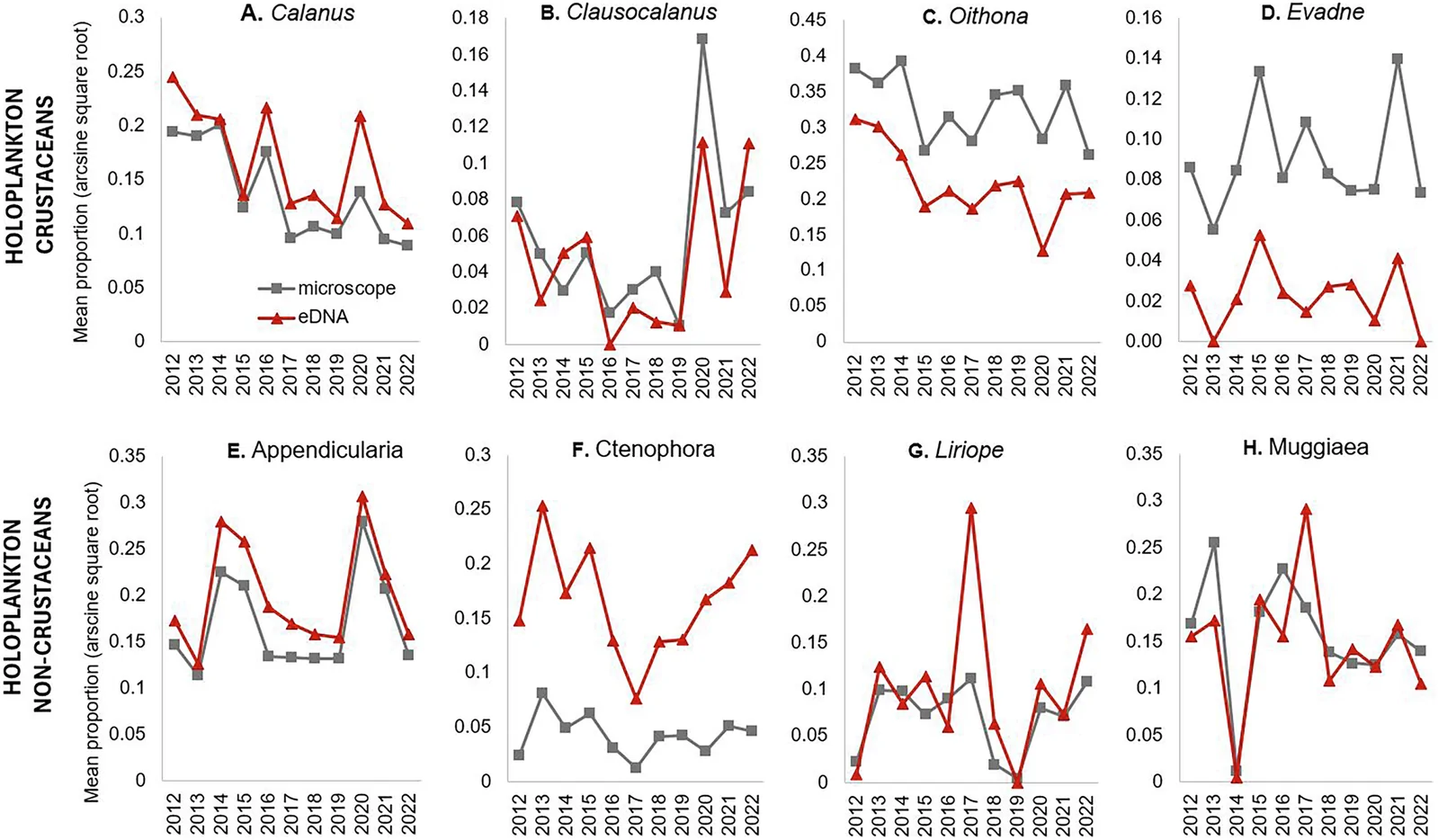
Seasonal patterns of selected zooplankton taxa identified by microscopy and eDNA. Comparisons of proportional annual average for microscope-derived abundance data (grey squares) and eDNA relative sequence abundance data (red triangles) for selected holoplankton **A**–**D** and non-crustacean holoplankton **E**–**H** (*n* = 11). For Appendicularia, sequence abundance is compared to microscope-derived biomass data as this was a better match than abundance data (Supplementary Fig. 1).

Spearman correlation tests to determine if eDNA- and microscope-derived data show similar patterns of occurrence across the time-series, first using monthly averages for each year (*n* = 124) to retain seasonal structure and allow comparison of recurring intra-annual patterns. This indicated that for 20 matching holoplanktonic crustacean taxonomic groups, 14 had significant correlations (Fig. 1c; Supplementary Fig. 1), despite differences in sampling depth and the properties quantified by the two methods. Taxa with significant correlations tended to be those that had higher abundance, with a significant relationship between the microscope/molecular time-series Spearman correlation value and the annual average abundance (derived from microscope counts) (R^2^ = 0.347; *p* = 0.01). This is consistent with both methods being more sensitive to common and biomass-dominant taxa. To assess the concurrence of longer-term trends, correlations were also calculated using annual average eDNA and microscope data (spanning 2012–2022). Despite having only 11 data points for comparison, annual average abundance data matched in their relative temporal patterns for eight holoplanktonic crustacean species (*Acratia*, *Calanus, Centropages, Clausocalanus, Temora, Oithona, Ditrichocorycaeus* and *Evadne* (Supplementary Fig. 1). Figure 2 compares annual average relative sequence abundance data with abundance data for three copepod taxa and *Evadne* sp., highlighting that the molecular time-series data based on surface water eDNA sampling can capture the key trends in the major taxa that are revealed by otherwise more expensive and time-consuming net sampling and microscope analysis.

Monthly resolution occurrence patterns also matched for Gelatinous and Semi-gelatinous Predators including Medusae (*Lizzia, Obelia*, *Liriope, Solmaris, Aglantha*), Siphonophorae, Ctenophora, Chaetognatha, and for Doliolida, Pteropoda and Appendicularia (Fig. 1C; Supplementary Fig. 2). Comparing annual average data shows eight taxonomic groups produced significant correlations: the Medusae *Lizzia*, *Liriope*, *Solmaris* and *Aglantha*, the Siphonophorae *Muggiaea* sp., Ctenophora, Chaetognatha, Doliolida and Appendicularia (Supplementary Fig. 2). Plots for Appendicularia, Ctenophora, *Liriope* sp. and *Muggiaea* (Fig. 2) highlight the matching annual trends. However, it must be acknowledged that the proportional contributions produced by the two methods differ.

For meroplankton, matching monthly resolution occurrence patterns were found for six of the 14 meroplankton groups (Anthozoa, Bivalvia, Cirripedia, Echinodermata, Nemertea, and Phoronida), but trends matched only for Phoronida annual average data (Supplementary Fig. 3). Given that the two approaches sample different parts of the water column and quantify different properties of the plankton community, this reduced correspondence likely reflects methodological complementarity rather than inconsistency.

#### Changes to zooplankton community composition differ among feeding mode

**Fig. 3:**
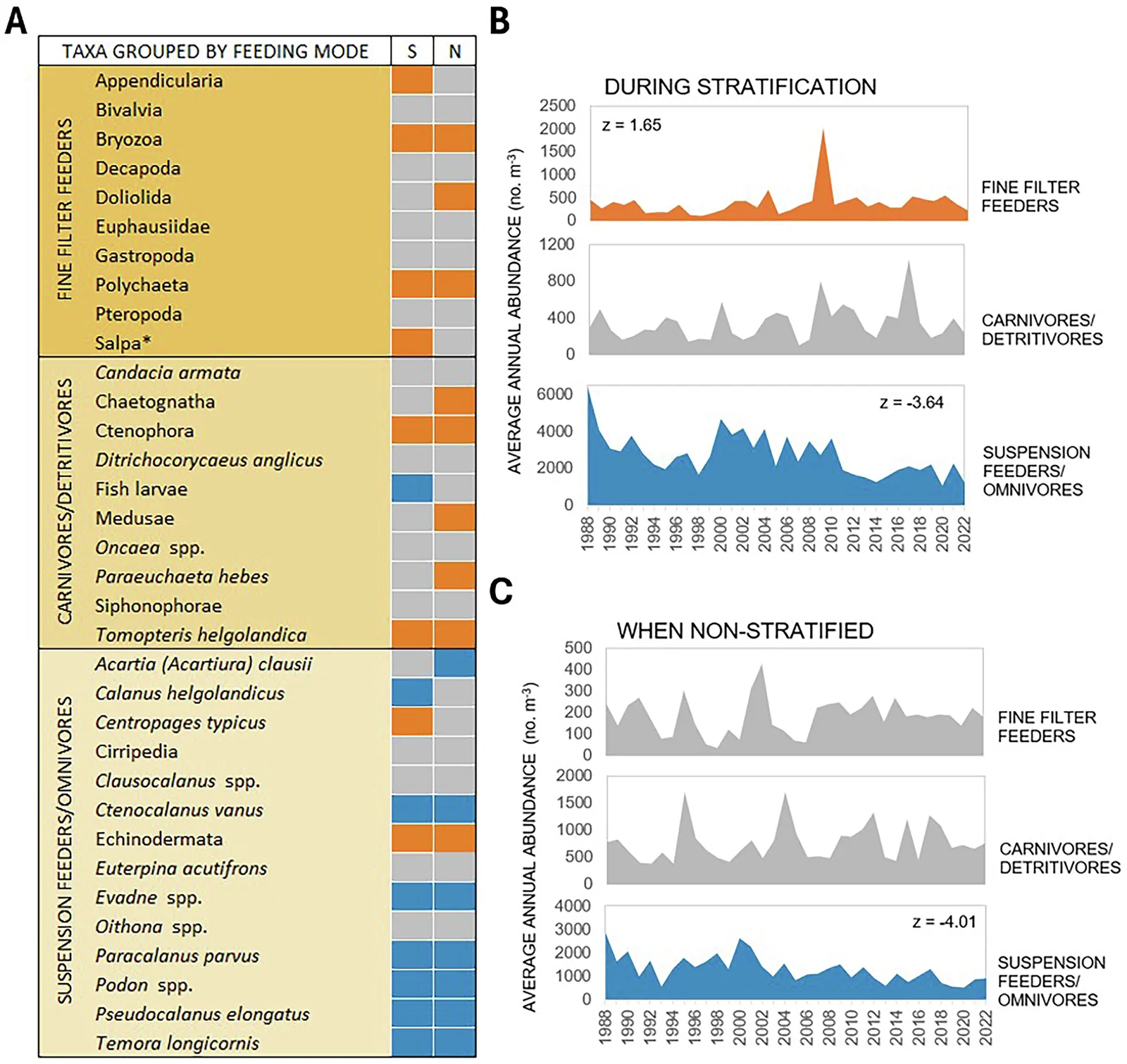
Long-term changes in zooplankton functional composition during stratified and non-stratified periods. **A** Trends in zooplankton feeding groups (Supplementary Table 2) derived from the morphological dataset (1988–2022). Annual average abundances were calculated separately for stratified (May – August) and non-stratified periods, and monotonic trends explored using the Mann Kendall test. Orange = significant increase over time; blue = significant decrease over time; Grey = no significant trend. Salpa (*) were first observed at L4 in 2022, and although trends are not significant, are included to illustrate recent seasonal occurrence. **B**, **C** Summary responses of functional groups. Fine filter feeders increased during stratification, while suspension feeders/omnivores declined. Mann Kendall Z values are shown for significant trends (*n* = 34).

Using the entire microscope time-series (1988-2022), the Mann Kendall test was applied to annual average abundance data to identify monotonic increases or decreases to the annual average abundance of 34 dominant zooplankton taxa (Fig. 3). Significant temporal trends were found when the species were classified by their feeding mode (Supplementary Table 2) and the year was divided into stratified periods (May– August) and the rest of year to account for seasonal differences in water-column structure, primary production, and nutrient availability, all of which influence zooplankton feeding conditions and trophic interactions^11^. During the former period, the abundance of fine filter feeding taxa (i.e. taxa that can catch particles down to a few microns in size) tended to increase (Fig. 3). This group includes Appendicularia, and the meroplankton taxa Bryozoa and Polychaeta (Fig. 3). Also included in Fig. 3 are Salpa. These were not detected at station L4 prior to 2022 but now appear regularly in summer. Regardless of whether the water column is stratified or non-stratified, the annual average abundance of several crustaceans has declined. This includes *Calanus helgolandicus*, *Ctenocalanus vanus*, *Evadne* spp., *Paracalanus parvus*, *Podon* spp., *Pseudocalanus elongatus* and *Temora longicornis* (Fig. 3). These taxa are classed here as omnivores because they are inefficient at retaining the smallest particles and do not target the larger, more motile cells as do the true carnivores.

Where possible, all eDNA-derived ASVs were also assigned a feeding mode (Supplementary Table 2). Although the eDNA time-series is considerably shorter than the morphological time-series, the eDNA time-series also showed a matching increase in annual relative sequence abundance of fine filter feeding taxa when the water column is stratified (Z = 1.87; *p* = 0.031).

### eDNA provides enhanced insight into patterns associated with meroplankton increases over time

**Fig. 4:**
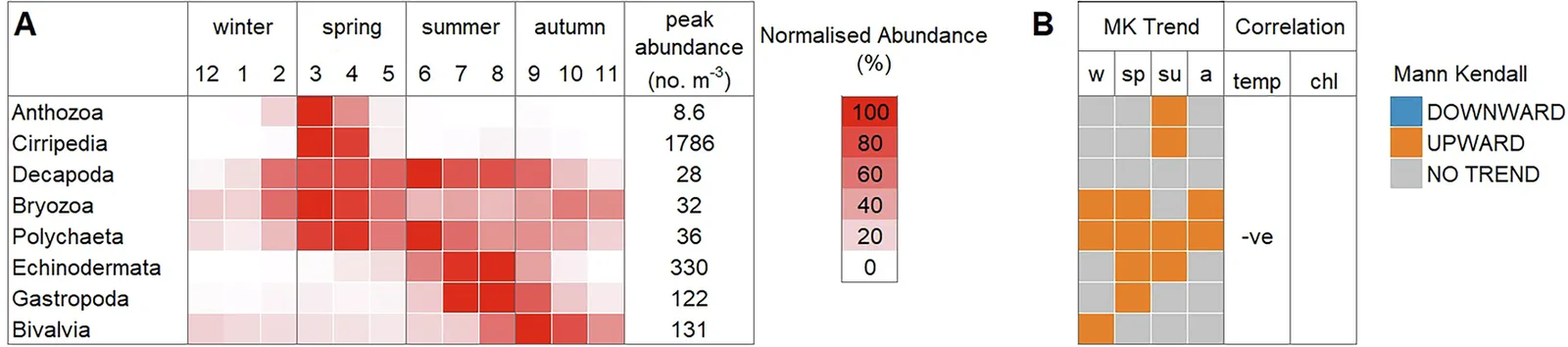
Seasonal patterns and long-term trends in major meroplankton groups. **A** Seasonal patterns of major meroplankton groups derived from the morphological timeseries (1988–2022). Abundances were standardised to the maximum observed value for each group to allow comparison among taxa; peak abundances (no. m^-3^) are indicated. **B** Significant long-term monotonic trends identified using the Mann Kendall tests. Significant Spearman correlations with temperature and chlorophyll concentration (using de-trended data) are also shown (*n* = 34).

Seasonal patterns and significant upward monotonic trends in the L4 1988 – 2022 time-series of zooplankton microscope data are shown in Fig. 4. The abundance of Bryozoa (z = 2.5; *p* = 0.006), Echinodermata (z = 2.05; *p* = 0.02) and Polychaeta (z = 2.3; *p* = 0.011) have increased over time (Mann Kendall test; *n* = 34). When seasonal trends are explored, the abundance of all meroplankton groups have increased within at least one season, except for Decapoda (Fig. 4). Using de-trended data, no positive correlations between annual average microscope-derived abundance of meroplankton groups and increasing temperature or decreasing chlorophyll measurements were found. For Polychaeta, de-trended data showed a negative relationship with temperature.

The higher taxonomic resolution provided by molecular methods offers the possibility to enhance our understanding of underlying mechanisms driving the increase to meroplankton groups. Most Animalia ASVs were Polychaeta (23.7% of all ASVs), but these Polychaeta ASVs only represented 1.4% of all Animalia sequences. Closely following Polychaeta in terms of sequence abundance were Ascidiacea (1.3%) and Echinodermata (1.3%) (Supplementary Fig. 1).

**Fig. 5:**
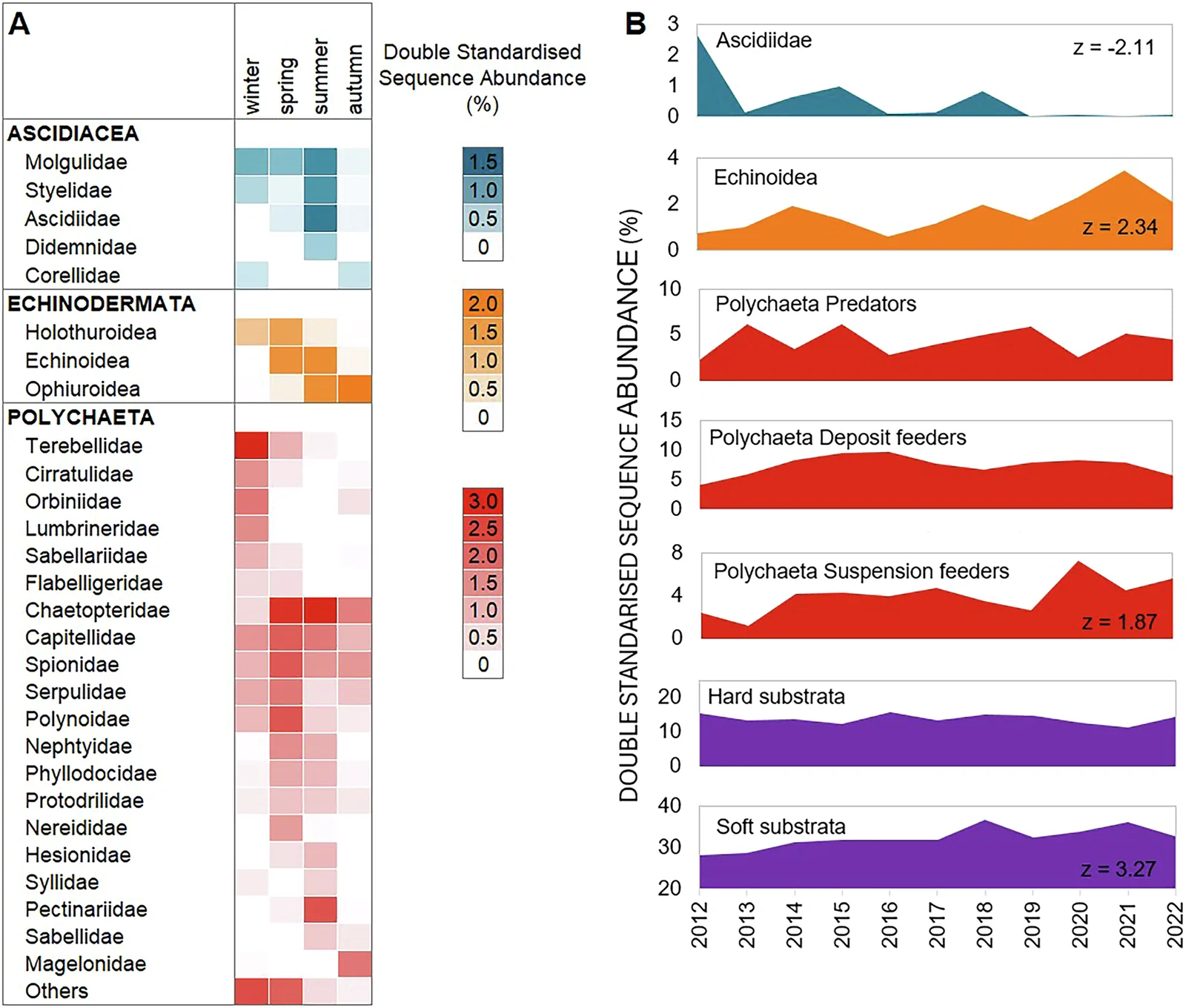
Seasonal and long-term patterns in meroplankton taxonomic and functional groups. **A** Average seasonal relative sequence abundance of selected meroplankton taxonomic groups (Ascidiacea, Echinodermata and Polychaeta). **B** Long term trends in relative sequence abundance for representative taxa and functional groups. Ascidiidae showed declining relative sequence abundance, whereas Echinoidea increased over time. Polychaeta ASVs were grouped according to inferred adult feeding mode (predator, deposit or suspension feeder; in red), and all meroplankton ASVs were grouped according to adult habitat type (hard or soft, in purple). Temporal trends in annual relative sequence abundance were assessed using the Mann Kendall tests; the corresponding Z value shown on the plot (*n* = 11).

Sequence data shows that polychaeta meroplankton are taxonomically diverse and exhibit marked seasonality, with different taxa peaking at different times of the year (Fig. 5). All meroplankton ASVs were assigned to adult habitat (*e.g*. soft or hard substrata) and adult feeding group (*e.g*. deposit feeder, suspension feeder or predator) based on the inferred taxonomic identity of the ASVs and where possible (Supplementary Table 1). Over the 11-year eDNA time-series, annual relative sequence abundance of meroplankton produced by adult suspension feeding Polychaeta showed an upward temporal trend in relative sequence representation (Mann Kendall test: Z = 1.87; *p* = 0.031; *n* = 11 annual values), predominantly due to increases in these meroplankton in spring months (Mann Kendall test: Z = 1.71; *p* = 0.043).

Our morphological time-series has shown Echinodermata to be increasing in abundance during spring and summer. eDNA data shows a seasonal succession with Holothuroidea (sea cucumbers) appearing first, followed by Echinoidea (sea urchins) and Ophiuroidea (brittle stars) (Fig. 4B). Over the 11-year eDNA time-series, the annual relative sequence abundance of Echinoidea showed a significant increasing trend through time in relative sequence contribution (Z = 2.34; *p* = 0.01; Fig. 4B).

Although Ascidiacea are rarely present in the L4 microscope dataset, the molecular data indicates Ascidiacea meroplankton to occur more prominently in the eDNA data (Supplementary Table 1). The molecular data show diverse Ascidiacea to be present in the water column, dominated by the sea squirts Molgulidae, Ascidiidae and Styelidae with Molgulidae in higher abundance in the water column through winter to summer months, Ascidiidae in summer months and Styelidae from spring to autumn months (Fig. 3b). However, over the 11-year eDNA time-series the relative sequence abundance of Ascidiidae has significantly decreased (Mann Kendall test: Z = -2.11; *p* = 0.018).

When all meroplankton are grouped according to adult habitat (i.e. predominantly residing either on hard substrata or are known epiphytes; living within or on soft sediments; or known to inhabit both hard and soft substrata; Supplementary Table 1), the relative sequence abundance of meroplankton from soft habitats showed a significant increasing trend in relative contribution over time (Fig. 3c). This group was dominated by Polychaeta, but included Phoronida, Platyhelminths, Bivalvia, Echinodermata, Mollusca and Nemertea (Supplementary Data 1).

## Discussion

The long-term changes seen at the Plymouth L4 site reflect much more widespread patterns documented across large stretches of both shelf and oceanic waters fringing the NE Atlantic^5,15^. The warming of these regions over the last 60 years has been associated with a major decline in holoplankton, mainly during the summer months^11^ and roughly a doubling of meroplankton abundance^2^. These changes are consistent with a reorganisation of pelagic food webs and potentially a shift in the strength of benthic-pelagic coupling whose causes are still under debate^8,20^. However, we were unable to link changes in the abundance of meroplankton simply with increasing temperature (Fig. 4a). For Polychaeta, de-trended annual average data showed a negative trend with temperature, indicating that the observed increases in polychaeta meroplankton are not simply or directly explained by temperature alone. Likewise, Continuous Plankton Recorder (CPR) data from across the NE Atlantic from 1993 to 2021 showed that meroplankton abundance declined above a temperature of 12.5 °C and instead, changes to the strength of stratification were a more important driver, with meroplankton abundance also higher in shallower waters^15^. Although only a relatively short time span of 11 years, our molecular time-series provided compelling evidence that the relative contribution of meroplankton from adult species that reside in soft sediment habitats rather than on hard substrata has increased (Fig. 4b). This suggests that increases to meroplankton abundance are unlikely to originate solely from changes occurring in the littoral zone or through any increase in the availability of man-made structures^21^ that provide more habitat for benthic species that require a hard substrate.

Instead, feeding mode emerged as a common trait distinguishing taxa showing contrasting long-term trends. The main taxa that declined were the omnivores, typified by suspension feeding copepods that rely on capturing relatively large ( > 5 µm) individual cells including bloom-forming diatoms, dinoflagellates, or ciliates and micrometazoa^22^. These 5-200 µm cells captured by the omnivores showed substantial declines both at L4 and more widely^2,19^ with an increase in relative dominance of picoplankton, particularly in summer^11^. In contrast, the holoplankton taxa showing increasing trends were predominantly fine filter feeders, which may benefit from a greater availability of smaller pico-size cells.

A similar pattern is evident for meroplankton, where the taxa showing increasing relative sequence representation included a broad range of taxa detected with sediment-dwelling adult stages (e.g. Bivalvia, Bryozoa, Decapoda, Echinodermata, Gastropoda, Nemertea, Phoronida, Platyhelminthes and Polychaeta). A common characteristic of many of these taxa is that their meroplanktonic larvae are known to function as fine filter feeders (Fig. 3a). Taken together, these patterns are most consistent with changes in stratification and nutrient supply, acting indirectly through the base of the food web to favour small primary producers and their grazers. While we cannot rule out modulating effects from changing fishing patterns or top-down control, the changes observed span very large scales suggestive of climatic forcing^2^. Under this interpretation, the observed increases in meroplankton abundance may primarily reflect changes in water column processes rather than direct benthos responses and may represent occupancy of ecological space associated with declining suspension-feeding copepods.

Understanding changes in meroplankton is challenging, since they have complex and potentially interacting drivers from both the benthic and pelagic parts of their life cycle. Close examination of Polychaeta taxa as revealed through eDNA show that the relative sequence representation of meroplankton from suspension feeding adult Polychaeta have increased through time (Fig. 4B). However, interpretation of eDNA-derived meroplankton signals requires caution. Both larvae and eDNA can be transported vertically and horizontally by advection, mixing and stratification, potentially decoupling detection from sites of larval origin or adult residence. Larval durations vary widely among taxa^23^, and the relationship between larval occurrence and adult habitat or feeding traits is not always straightforward, as some taxa can occupy mixed habitat ranges^24^. Consequently, eDNA-based trait assignments should be regarded as indicative rather than definitive. Notwithstanding these uncertainties, the observed shifts in meroplankton sequence composition remain consistent with broader changes in pelagic food-web structure. Because production in the water column fuels the benthic food web, changes to the structure of pelagic food webs (e.g. plankton abundance, community composition, phenology and physiology) could also impact the flux of fresh organic matter to benthic habitats. Consequently, the composition and ecosystem function of benthic communities could also be expected to adapt. More attention is therefore warranted to understanding how potential changes to the fate of phyto-detritus result from changes in food web structures^11^. It is conceivable that these changes could influence the structure and function of the benthic community, in turn altering both the regeneration of nutrients for primary production, and the availability of benthic fauna for pelagic consumer predation.

Our study adds to the rapid expansion in the use of eDNA to monitor ecosystems, both on land and in the sea^12^. However, it also illustrates the insights that are only possible by combining eDNA with microscope-based methods. Microscope-derived data typically benefit from much longer time series than eDNA^25^ and in our study this provided the statistical power to detect long-term changes related to broad feeding traits. The eDNA provided a shorter time series but greatly improved taxonomic resolution, enabling us to determine which meroplankton taxa were being increasingly advantaged.

A second benefit of our eDNA sampling is that the 415 parallel samples analysed with eDNA and microscopy over 11 years allows the essential calibration needed to better assess the strengths and weaknesses of this rapidly expanding sampling method. Our eDNA sampling was very simple surface water sampling, enabling it to be achieved with minimal logistics, and so was never intended as a rigorous cross comparison with the full depth net sampling. Nevertheless, we show that trends in many of the key, abundant and biomass-dominant taxa agreed remarkably well between the two methods (Figs. 1C and 2). Thus, eDNA with a simple water collection method not requiring a research ship could be useful for discerning trends for key taxa, including copepod genera that dominate zooplankton communities globally (*e.g. Acartia*, *Calanus*, *Clausocalanus*, *Oithona*, *Paracalanus*, and *Pseudocalanus*). This highlights the possibility that molecular techniques can be used to monitor the key bioindicators of rapid change.

Clearly, eDNA methods also carry a suite of limitations. Our results show that the 18S rRNA V9 marker cannot replicate the species-level of detail provided by a trained taxonomist (Supplementary Table 1). Although the 18S rRNA V9 marker used here identified a broad range of taxonomic groups (spanning 15 Phyla), this was at the expense of lower taxonomic resolution, with the level of detection varying according to taxonomic group (detailed in Supplementary Data 1 and Supplementary Table 1). For example, Copepoda groups can be identified to Genus level with confidence, but other groups can only be identified to Family (e.g. Appendicularia, several Medusae), Order (e.g. Anthozoa and Bivalvia) or even Class (e.g. Echinodermata). Although COI metabarcoding can provide finer species-level resolution, its application to eDNA from zooplankton communities is confounded by primer bias and variable amplification success across taxa^26^. A multiple marker metabarcoding approach would be expected to improve taxonomic resolution^27,28^, such as the COI^28–31^ in combination with an 18S marker, thereby allowing known gaps for the COI marker (e.g. certain Copepoda, Appendicularia and Thaliacea^26^ to be covered by an 18S marker.

Several studies have concluded that molecular-derived zooplankton data can produce similar relative abundance of taxa to those produced by morphological analyses, often with results more comparable to biomass data^13,27,31,32^. Our eDNA data provided a different perspective on zooplankton composition when compared to microscope-based abundance or biomass data (Fig. 1). There are several reasons why the proportional representation of taxonomic groups does not match. Firstly, our minimalist surface sampling approach for eDNA would be expected to detect a different range of taxa to the full depth net sampling. For example, euphausiids and Salps were present in net samples but missing in the eDNA data. Both can aggregate and perform diel vertical migrations to “hide” in lower light levels nearer the seabed during the daytime when all the sampling was conducted). Likewise, there is evidence that despite a few neuston-specialists, most zooplankton tend to avoid the top few meters of the water column possibly due to turbulence or high UV radiation^33,34^. Secondly, fragile gelatinous zooplankton including ctenophores^35^ and appendicularians^36^ have been shown to be underrepresented in net samples, with some evidence to suggest that Ascidiacea may also be underrepresented^37^. Thirdly, even if our eDNA sample were derived from the net sample, the translation of molecular-derived metabarcode data to actual abundance or biomass would still not be straight-forward. Technical biases can be introduced at every step in the creation of a molecular data set, each of which can impact the final data obtained; this includes both temporal and seasonal fluctuations in the longevity of eDNA within water^16,17^ and methodological choices associated with eDNA data generation^18^. High gene copy numbers for certain taxa or markers could also inflate relative sequence abundance in comparison to actual abundance or biomass^38^. However, the matching trends shown for key holoplankton crustaceans and gelatinous taxa (Fig. 3), offers the possibility of creating algorithms to transform relative sequence abundance counts to more closely approximate abundance and/or biomass.

Our detailed method comparison highlights that eDNA-derived data can provide a valuable alternative, sometimes very different picture of zooplankton dynamics. We should continue to explore the capabilities and limitations of eDNA datasets to understand their full potential, but this should not be at the expense of standard monitoring^25^. Our results clearly show that molecular data cannot replicate morphological data, rather there are merits to both methods, and that they should be considered complementary. Molecular time series are not so long as those for microscope-based time series so do not have the statistical power to properly understand how plankton are responding to various pressures. For this reason, they need to be run alongside traditional time series to intercalibrate them. More existing monitoring programmes should consider pairing new methods while maintaining existing time-series data collection using morphological identification.

## Methods

### Zooplankton microscope dataset

Weekly vertical hauls (0–50 m) have been taken at station L4 (50°15.00’N, 4°13.02’W; depth ~55 m) using 200 µm WP2 nets since March 1988. Two replicate samples are enumerated and identified by a trained zooplankton taxonomist and the results then averaged to provide a single mean value per timepoint^39^. Carbon biomass was estimated by converting site- and season-specific length measurements to carbon using literature-based conversion factors as detailed in McEvoy et al.^40^. Abundance is expressed as individuals per m^3^ and biomass as mg C per m³. Feeding traits (fine filter feeders, suspension feeders/omnivores, carnivores/detritivores) were assigned to 33 key taxa based on published literature (Supplementary Table 2).

### Zooplankton eDNA dataset

Between February 2012 and March 2023, weekly surface seawater samples (5 L) were collected at station L4 (415 dates), filtered through 0.22 mm Sterivex cartridges, flash-frozen in liquid nitrogen, and stored at −70 °C. Nucleic acids were extracted using an AllPrep DNA/RNA Kit (Qiagen), with the following modifications. RLT buffer (700 µL) was added to the Sterivex, vortexed for 2 min, sonicated on ice for 10 min, and then incubated for 30 min at 65 °C in a rotating hybridisation oven. The lysate was removed from the Sterivex and set to one side while a second 700 µL aliquot was added to the Sterivex, and the process of vortexing, sonication and heating repeated. Once the second aliquot was added to the first aliquot, the AllPrep DNA/RNA Kit protocol was followed according to the instructions of the manufacturer. Purified nucleic acids were stored at -70 °C. Quality and quantity of DNA were assessed using a Nanodrop spectrophotometer.

PCR and sequencing were performed by Nu-OMICS (Northumbria, UK). The 18S rRNA V9 region was amplified with primers Euk_1391F (GTACACACCGCCCGTC)^41^ and EukBr (TGATCCTTCTGCAGGTTCACCTAC)^42^. This marker was selected as it provides broad amplification across metazoan zooplankton^43,44^. While COI markers can offer higher species-level resolution for some taxa, they are known to exhibit strong primer bias and inconsistent amplification of key zooplankton groups, including Copepoda, gelatinous taxa and meroplankton^26^. Given the aims of this study (to assess the relative temporal patterns and functional traits across the community), the 18S V9 marker was considered the most appropriate choice.

Eukaryote profiling of the V9 marker was carried out using the method described to the Earth Microbiome Project^45^. PCR was carried out using 1x KAPA2G Hotstart Robust MasterMix, 0.2 µM each primer and 1 µl of template DNA in a total volume of 25 µl under the following conditions: 94 °C for 3 min, 35 cycles of 94 °C for 45 s, 57 °C for 60 s and 72 °C for 90 s with a final extension at 72 °C for 10 min. DNA kit extraction controls, along with one positive and one negative PCR controls were included. PCR products were normalised using a Quant-iT™ PicoGreen™ dsDNA Assay Kit (Invitrogen) as described in the manufacturer instructions and each 96 well plate was pooled in equimolar amounts per sample on a Hamilton MicroLab Star. Each pool was cleaned using 1:1 Ampure XP:Pooled amplicon, fragment size checked by BioAnalyzer (Agilent Technologies) and concentration by a Qubit™ 1X dsDNA High Sensitivity assay kit (Invitrogen). Sample pools were combined in equimolar amounts to create a single 4 nM library then denatured using 0.2 N NaOH for 5 min and diluted to a final concentration of 5 pM, supplemented with 20% PhiX and loaded onto an Illumina MiSeq using the V2 500.

Demultiplexed paired-end FASTQ files were processed in QIIME2^46^ with ASVs generated using DADA2^47^. The short V9 region ( ~ 130 bp) enabled full overlap of reads, and sequences with quality scores <30 were removed. Taxonomy was assigned using Naïve Bayes classifiers trained on SILVA 138^48^ and PR2 databases^49^, and results were cross-checked with BLAST against the latest NCBI nt database (downloaded June 2024).

Sequences identified as Animalia were aligned and compared to reference Animalia DNA sequences (downloaded from Genbank), and neighbour-joining phylogenetic trees with 100 bootstrap replications were generated in MEGA11^50^. Each ASV was manually checked against database sequences and the L4 microscope dataset to determine the limits of taxonomic resolution. This process identified 427 unique Animalia ASVs (totalling 1,494,588 sequences). This alignment and comparison of ASVs with sequences in DNA reference databases often resulted in a lower taxonomic level being chosen for an individual ASV. PML taxonomists provided expert input to match IDs to local species. Final taxonomic assignments (PR2, SILVA, NCBI vs manual QC), including representative sequences for each ASV are detailed in Supplementary Data 1.

When comparing eDNA and microscope datasets, taxa were grouped as Crustaceans (Branchiopoda, Calanoida, Cyclopoida, Canuelloida and Harpacticoida), Meroplankton (Anthozoa, Ascidiacea, Bivalvia, Bryozoa, Cirripedia, Decapoda, Echinodermata, Gastropoda, Nemertea, Phoronida, Platyhelminthes and Polychaeta), Gelatinous Zooplankton (Medusae, Siphonophorae, Ctenophora and Chaetognatha, plus Hemichordata and *Tomopteris*), and Others (Appendicularia, Doliolida and Pteropoda).

### Comparison of datasets and statistics

For this study, only samples matching the molecular time-series (16 Feb 2012–15 Mar 2023; 415 dates) were compared. The eDNA dataset was Wisconsin-standardised, and microscope-derived abundance and biomass data were converted to proportional data to allow direct comparison. Comparisons were made with both abundance and biomass data because eDNA sequence data do not translate directly to organism counts and may align variably with numerical versus size-weighted (biomass) representations across taxa^29^. Occurrence data were expressed as proportions and transformed using an arcsine square root transformation to reduce skewness and unequal variance. Congruence between molecular and microscope-derived time-series was assessed using Spearman rank correlations applied to monthly averaged proportional data to test for shared temporal patterns. Spearman correlations were chosen because they do not assume linear relationships and are robust to deviations from normality common in long-term ecological time-series. To reduce the influence of short-term temporal autocorrelation while retaining seasonal structure, correlation analyses were first conducted using monthly averaged data. Annual average proportional data were then used in separate Spearman correlation analyses to assess the concurrence of longer-term trends. Here, we used eDNA spanning 2012–2022 only.

Monotonic temporal trends within the annual average proportional data were also evaluated using Mann-Kendall tests (Minitab), a non-parametric approach suitable for long-term ecological time-series that are non-normally distributed and may contain outliers. Annual average temperature and chlorophyll data (data available to download from https://www.westernchannelobservatory.org.uk/) were compared to meroplankton abundance. A simple linear de-trending approach was applied prior to correlation analyses with temperature and chlorophyll to remove shared long-term trends and reduce the risk of spurious correlations. Where possible, meroplankton ASVs were assigned to their adult habitat (soft or hard substrata) and to their adult feeding group (deposit feeder, suspension feeder, predator) using BIOTIC^51^, WoRMS^52^, and Trait Explorer^53^. Taxa were excluded where trait information was insufficient or too ambiguous to assign confidently. Where variable results were reported, assignments reflect the most likely or ecologically relevant adult trait reported.

### Reporting summary

Further information on research design is available in the Nature Portfolio Reporting Summary linked to this article.

## Supplementary information

Transparent Peer Review file

Supplementary Information

Description of Additional Supplementary files

Supplementary Data 1

Supplementary Data 2

Reporting Summary

## Data Availability

All data used within this study are fully accessible. The microscope zooplankton time-series data are available via the British Oceanographic Data Centre (BODC; see^19^ to download). Data used to generate Figs. 1a, b, 2, 3 and 4 can be found in Supplementary Data 2. Raw sequence data have been deposited in the NCBI Sequence Read Archive under BioProject ID PRJNA1045854. In addition, details of Animalia ASVs identified in this study, including a representative sequence, taxonomic classification together with the ASV table are within Supplementary Data 1. This Animalia-specific ASV table was used to generate Figs. 1, 2 and 5. Refer to the supplementary data 2 file for data used to generate Figures.
