# Supplementary material for: eDNA and microscopy reveal traits underlying long-term zooplankton community change
Source: Commun Biol. 2026 Oct 27;9:1409. doi: 10.1038/s42003-026-10901-w (PMC13714307; doi:10.1038/s42003-026-10901-w)
Supplement: Supplementary file 1 [file 42003_2026_10901_MOESM1_ESM.pdf]

nature portfolio

# Peer Review File

# eDNA and microscopy reveal traits underlying long-term zooplankton community change

Corresponding Author: Dr Karen Tait

**This file contains all reviewer reports in order by version, followed by all author rebuttals in order by version.**

**Attachments originally included by the reviewers as part of their assessment can be found at the end of this file.**

Version 0:

Reviewer comments:

Reviewer #1

(Remarks to the Author)
Brief summary of the manuscript
The MS studies the plankton community in the English Channel based on an 11-year data-time series collected from a station ca. 30 km south of Plymouth (U.K.). What makes this investigation especially interesting is that it combines hydrographical data (environmental variability) and taxonomic data (plankton net samples) with data obtained from environmental DNA (eDNA) samples taken in surface waters. In total 425 samples contain information from both nets and eDNA. This allows for an approach that provides a broader picture of the plankton community than studies that rely only on net samples, only, and allow the authors to demonstrate that the two approaches vary considerably for some groups of zooplankton while being comparable for other groups. The authors claim that their approach allow for "a more holistic view of the structure, seasonality and trends in the plankton, to gain a better taxonomic appreciation of species that are increasing or decreasing" (l.64-65). The additional taxonomic information provided by the eDNA-data enable the authors to link trends in the data to groups of organisms and, by consulting literature on feeding modes, even to trait-based groups. They find that meroplanktonic forms of soft-sediment fauna and especially fine filter feeders increased in (relative) abundance during the study period.
Overall impression of the work
The ms is clearly interesting, providing new knowledge to the seasonality and between-year variability in the plankton community. They speculate that the observed community changes are in relation climatic impacts linked to declining nutrient supply and a concurrent shift towards smaller size-structure in the phytoplankton community and affect the benthic-pelagic coupling. This is a bit speculative, I think. However, using this framework to classify groups increasing in (relative) abundance as "climate winners" (or losers) both in the title and elsewhere (e.g. .22, 41, 42, etc.) is a bit misleading and not needed to identify species or groups that increase or decrease in apparent abundance between seasons and years based on a time-series analysis over a relatively short period (11 y). Judging by results presented in Fig. 3 and 4 it seems likely that what groups could be assigned to the winning and losing fractions is quite sensitive to the time periods of investigation (start the analysis two years later or end it two years earlier and you are likely to get different results). Claims about trends in the data related to climatic forcing therefore seems to me to be somewhat speculative long shot, given the relatively limited data set. I think the paper would benefit from an analysis of how seasonal variability and between year variability adds to the complexity and dynamics of plankton communities and would make for a very interesting, and quite possibly much cited, paper.
I cannot validate the molecular parts of the methodology; however, the statistical analysis seems valid and appropriate and all methods should be possible to replicate (provided access to data and similar samples).
Specific comments, with recommendations for addressing each comment
1. The end of the Introduction chapter (l.66 -72) seems to me to be stating and discussing own results somewhat prematurely. I suggest moving these statements to the Results- and Discussion chapters.
2. l.164. the stratified period is defined as April – August, but in l.186. it is from May – August. Probably a simple mistake, but quite confusing when trying to interpret the results in Fig. 3, and relating to results presented in Fig. 4, where seasons and months are used instead.
3. In Fig. 4. I struggle to understand what criteria was used in the selection of taxonomic and/or functional groups from B) that is shown in C). Please provide more information in figure legend!
4. In Methods (l. 371-373) I find it a bit unclear why they have included biomass as it seems to contribute little to the main

story, but necessitates a longer section (l. 329 – 353) that mainly deals with providing explanations for why their eDNA-approach does not match the biomass data. I find this more confusing than aiding in clarifying the main results. While it is interesting that the results deviate from other similar studies this may be an additional topic, perhaps better approached by a separate study. Thus, I do not see that the analysis benefit much from adding biomass data and recommend cutting the biomass part altogether from the presentation.
5. Also (l.373 – 374) why report abundance values of matazoans as "cells per millilitre" instead of individuals per volume unit?

Reviewer #2

(Remarks to the Author)
Brief summary of the manuscript:

This manuscript compares long-term zooplankton data obtained from traditional morphological identification of samples collected using 200 µm WP2 nets against an 11-year eDNA time series data (2012 – 2023) at Station L4 in the Western English Channel. Using both morphological and molecular approaches, this manuscript aimed to examine taxonomic trends and functional traits of zooplankton "winners and losers" under climatic warming.

The manuscript displayed strong agreement between both methods for dominant holoplankton taxa, while eDNA revealed higher taxonomic resolution for meroplankton. The results from this study suggest that increases in meroplankton are primarily associated with fine filter-feeding taxa originating from soft-sediment habitats, which are likely linked to indirect climate effects mediated through stratification, nutrient supply, and phytoplankton size structure rather than through temperature alone.

The manuscript is suitable for publication after minor revision (possibly this qualifies as major revision given the extent of minor edits required?)

Overall impression of the work:

This is a strong, timely, and carefully executed study and is highly significant to plankton ecology, marine food web dynamics, and climate change research. The manuscript clearly demonstrates that eDNA complements, rather than replaces, traditional monitoring based on microscope identification of zooplankton, and strengthens the argument for molecular-based approaches to monitoring biodiversity.

The manuscript is generally well-written, and the statistical analyses seem appropriate and easily reproducible with the same dataset. However, the manuscript contains a substantial number of minor errors (e.g. taxonomic misspellings, incorrect Figure or Table referencing, mismatch between text and data), which suggests the final checking was rushed. Please refer to the detailed list of comments attached.

Reviewer #3

(Remarks to the Author)
I co-reviewed this manuscript with one of the reviewers who provided the listed reports. This is part of the Communications Biology initiative to facilitate training in peer review and to provide appropriate recognition for Early Career Researchers who co-review manuscripts.

Reviewer #4

(Remarks to the Author)
please, see the file attached.

Version 1:

Reviewer comments:

Reviewer #1

(Remarks to the Author)
I have carefully evaluated the authors responses and changes made in the MS in response to my comments. I find the responses appropriate and have no further comments and thus suggests that the MS can be published in Communications Biology.

**Open Access** This Peer Review File is licensed under a Creative Commons Attribution 4.0 International License, which permits use, sharing, adaptation, distribution and reproduction in any medium or format, as long as you give appropriate credit to the original author(s) and the source, provide a link to the Creative Commons license, and indicate if changes were made.
In cases where reviewers are anonymous, credit should be given to 'Anonymous Referee' and the source.
The images or other third party material in this Peer Review File are included in the article's Creative Commons license, unless indicated otherwise in a credit line to the material. If material is not included in the article's Creative Commons license and your intended use is not permitted by statutory regulation or exceeds the permitted use, you will need to obtain permission directly from the copyright holder.
To view a copy of this license, visit https://creativecommons.org/licenses/by/4.0/

***Response to Reviewers comments***

**Tait et al: eDNA complements microscopy to identify shared traits of zooplankton winners and losers under climatic warming**

Now with the new title **eDNA complements microscopy to identify shared traits of zooplankton increasing or decreasing under climate change**

We would like to thank the reviewers for their time and careful evaluation of our manuscript. We are grateful for the thoughtful and constructive comments provided, which have helped us to improve both the clarity and overall quality of the work. We have considered all points in detail and have revised the manuscript accordingly, aiming to address each concern thoroughly. We believe that these revisions have strengthened the manuscript, and we appreciate the opportunity to resubmit an improved version to *Communications Biology*.

Given their detailed response, we first outline our response to reviewer 4, followed by reviewers 1 and 2. Line numbers refer to the marked changes version of the manuscript.

***Reviewer #4***

**Major comments**

**1. The causal interpretation is stronger than the evidence currently supports**

Please, moderate the causal language throughout the manuscript.

We thank the reviewer for this constructive comment. Following their suggestion, we have systematically revised the manuscript to moderate causal language and better distinguish observed patterns from mechanistic interpretation. These revisions are detailed in the table below.

| Line | Original | Change Made |
|---|---|---|
| Title (line 1) | *eDNA complements microscopy to identify shared traits of zooplankton winners and losers under climatic warming* | *eDNA complements microscopy to identify shared traits of zooplankton increasing or decreasing under climate change* |
| Abstract (line 22) | *eDNA revealed that the meroplankton increase was driven by soft sediment…* | *eDNA revealed a shift towards higher relative sequence abundance of soft sediment…* |
| Abstract (line 24) | *climate winners….* | *….the taxa showing increasing trends were mainly …* |
| Abstract (line 26) | *…suggesting that water column processes, mediated by declining nutrient supply …* | *…This suggests that changes in water-column processes, linked to declining nutrient supply and a shift towards smaller phytoplankton ….* |
| Introduction (line 44) | *Coarse taxonomic- and temporal-resolution, particularly of the meroplankton has prevented our understanding of the common traits shared by the winners and losers under climate change.* | *Coarse taxonomic- and temporal resolution, particularly of the meroplankton, has limited our ability to identify of the common traits among taxa showing contrasting trends under climatic change….* |
| Results (line 185) | **Title:** *Changes to zooplankton community composition are linked to feeding mode* | **Title:** *Changes to zooplankton community composition differ among feeding mode* |
| Results (line 229) | **Title**: *eDNA provides enhanced insight into drivers of* | **Title:** *eDNA provides enhanced insight into patterns associated* |

| | | |
|---|---|---|
| | *meroplankton increases over time* | *with meroplankton increases over time* |
| Discussion (line 312) | *These changes mark a profound reorganisation* | *These changes are consistent with a reorganisation of pelagic foodwebs* |
| Discussion (line 325) | *This suggests that increases to meroplankton is not driven from the seabed by changes occurring in the littoral zone….* | *This suggests that increases to meroplankton abundance are unlikely to originate solely from changes occurring in the littoral zone….* |
| Discussion (line 328) | *Instead, the common trait dividing the winners and losers under climate change appeared to be feeding mode…* | *Instead, feeding mode emerged as a common trait distinguishing taxon showing contrasting long-term trends….* |
| Discussion (line 342) | *The interpretation that best fits the data is that the changes were driven by indirect temperature effects on stratification and nutrient supply…* | *Taken together, these patterns are most consistent with changes in stratification and nutrient supply, acting indirectly through the base of the food web…..* |
| Discussion (line 347) | *We therefore suggest that the meroplankton increase reflected changes in the water column rather than the benthos and they were filling a niche left by declining suspension-feeding copepods …* | *Under this interpretation, the observed increases in meroplankton abundance may primarily reflect changes in water-column processes rather than direct benthic responses and may be occupying ecological space associated with declining suspension-feeding copepods…* |

**2. The comparison between microscopy and eDNA needs more careful framing**
A key issue is that the two methods are not directly equivalent. In addition, the outputs differ, microscopy provides abundance/biomass estimates, while eDNA provides sequence-based compositional data. The manuscript acknowledges some of this, especially in the Discussion but not strongly enough in the framing of the Results and Abstract.

Please, make this limitation much more explicit earlier in the manuscript, especially in the Abstract and Results. It should be clear that the methods sample different parts of the water column, they quantify different things, lack of agreement is not necessarily methodological failure, agreement is notable precisely because the methods are not equivalent. This will make the conclusions more credible.

Limitations of word count within the abstract means we are unable to include this information in the abstract.

We have added an additional sentence to the Introduction (**line 71**: *'Importantly, our eDNA- and microscope-based methods sample different components of the water column. They also quantify fundamentally different properties of plankton communities (e.g. sequence vs numerical abundance), such that exact correspondence between methods is not expected.'*

Within the results section, we have made the following additions:

- We have removed the sentence describing the higher number of Animalia ASVs found compared to the number of taxonomic units produced by microscope analyses (**Line 91).**

- **Line 124**: *This indicated that for 20 matching holoplanktonic crustacean taxonomic groups, 14 had significant correlations (Figure 1c; Supplementary Table 3),* we have added the statement '*despite differences in sampling depth and the properties quantified by the two methods'*.
- When describing the correlation between relative sequence abundance and the abundance of dominant Copepoda (**Line 132**), we have added the statement '*This is consistent with both methods being more sensitive to common and biomass-dominant taxa'*.
- We conclude (**Line 155**) '*Given that the two approaches sample different parts of the water column and quantify different properties of the plankton community, this reduced correspondence likely reflects methodological complementarity rather than inconsistency'*.

**3. The statistical framework needs many more details**
The analytical methods are currently under described relative to the weight placed on the conclusions. Important details are missing or not very clear yet.
Examples:
- monthly averages are used for some comparisons (**lines 109–113**), whereas annual averages are used elsewhere (**lines 116–120**, **160–164**, **198–205**). The reason is not fully explained.
- it is unclear whether temporal autocorrelation and seasonality were accounted for in the correlation analyses.
- there is no mention of correction for multiple testing despite many correlations and trend tests.
- the de-trending procedure used before testing temperature/chlorophyll relationships is not described (**lines 203–205**, **243–244**, **415–417**).
- the manuscript defines stratified periods inconsistently: "*April–August*" (**line 164**) versus "*May–August*" (**line 186**).
- it is not clear exactly what unit was entered into each Mann–Kendall test: annual averages? Seasonal annual averages? Proportions?
- in figure 1B (**line 136**), why the biomass doesn't reach 1 if it is relative contributions, like abundance and eDNA do?
Please, improve the statistical section (**lines 409–419**) to include exact temporal aggregation used for each analysis, sample sizes for each test, how seasonality and autocorrelation were handled, how de-trending was performed, whether multiple-testing correction was considered. Why Spearman, Pearson, and Mann–Kendall were chosen for specific comparisons? How stratified vs non-stratified periods were defined and why?

We have carefully reviewed Method descriptions throughout the manuscript and made the following changes:

- We have ensured greater clarity on why both monthly averages ('*to retain seasonal structure and allow comparison of recurring intra-annual patterns'* (**line 126**)) and annual average data ('*to assess the concurrence of longer-term trends'* (**line 134**)) and we have ensured that the reader is more aware of which dataset is used at each stage.
- Stratification occurs during May – August at Station L4. This has been amended (**line 190)** and an additional statement added to explain why the year was split into stratified and non-stratified periods (**line 191**: *'to account for seasonal differences in water-column structure, primary production, and nutrient availability, all of which influence zooplankton feeding conditions and trophic interactions'*).
- The methods section (**lines 504 – 533**) now contains substantially more detail on statistical methods, including how and why datasets were transformed, and why

Spearman correlations and Mann-Kendall tests were chosen. In all cases, the Mann-Kendall tests used annual average data (either microscope or eDNA), and we have made changes throughout the manuscript to ensure the reader is aware of this.

- The methods section also now describes the de-trending method used (linear; **line 526**).
- **Figure 1B** has been adjusted and replaced.

We agree that temporal autocorrelation and seasonality are important considerations in long-term plankton time-series. These were addressed by conducting correlation analyses on monthly averaged data rather than weekly observations, which reduces short-term serial dependence while retaining seasonal structure. In addition, analyses were stratified by season where appropriate. We have clarified this in the Methods.

We acknowledge the issue of multiple testing. However, the analyses were hypothesis-driven and the tests are not statistically independent, as taxa respond to shared environmental forcing. We therefore focused on coherent patterns across taxa and trait groups rather than isolated p-values, and have clarified this rationale in the manuscript.

**4. Trait assignments are important, but the framework is not transparent enough**
The main conclusion is that changes are linked to feeding mode, especially the distinction between fine filter feeders, suspension feeders/omnivores, and carnivores/detritivores (**lines 159–178**, **182–194**, **272–285**, **374–376**). However, the basis for assigning taxa to these categories is not sufficiently explained in the main text. Similarly, meroplankton are grouped by adult habitat (hard/soft/both) and by adult feeding mode (**lines 213–217**, **232–249**, **418–419**), but these assignments are ecologically complex and likely uncertain for some taxa.
Please, clarify whether feeding traits refer to larval stages, adult stages, or generalized taxon-level traits, how ambiguous taxa were handled, whether taxa with mixed ecology were excluded or forced into a single class, how robust the hard/soft substratum assignments are. This is important to the interpretation. I strongly suggest either adding a good explanation in the main text or adding a sensitivity analysis showing whether the key conclusions remain if ambiguous taxa are excluded. Important sections include **lines 66–72**, **159–178**, **213–217**, **232–249, and 374–376**.

Within the methods, we now clarify that we refer to the adult habitat and feeding group for meroplankton. Taxa were excluded where trait information as insufficient or too ambiguous to assign confidently. Where variable results were reported, the chosen assignment reflected the most likely or ecologically relevant adult trait reported (**line 531**).

**5. The "*no evidence for temperature-driven meroplankton increase*"**
The manuscript states in the Abstract that *"There was also no evidence that increases to meroplankton groups were driven by temperature"* (**lines 21–22**). Similar statements appear in the Results (**lines 201–205**) and Discussion (**lines 259–266**). This conclusion seems too strong. The analyses described appear to show only that de-trended annual average abundance was not positively correlated with temperature, and that polychaetes showed a negative relationship. That does not rule out nonlinear responses, seasonal effects, lagged responses, indirect temperature effects via stratification or food-web structure, taxon-specific responses hidden by aggregation.
Please, revise these claims to reflect what was actually tested. A more defensible wording would be something like *"We found no simple positive relationship between de-trended annual meroplankton abundance and temperature."* Think about it!

We have changed this in the abstract: (**line 23**) to '*We found no evidence for simple positive relationships with temperature but instead taxa showing long-term increases were mainly fine filter feeders, including many meroplankton*.

**6. The statement that increasing meroplankton derives mainly from soft-sediment taxa is interesting, but still inferential**
One of the most interesting contributions of the study is the suggestion that the increase in meroplankton is driven by taxa whose adults inhabit soft sediments rather than rocky nearshore habitats (Abstract **lines 19–21**; Results **lines 232–237**; Discussion **lines 267–271**). This is plausible and potentially important. However, the manuscript currently presents this with too much certainty. Potential complications include advection of larvae and eDNA, transport of DNA in the water column, uncertain mapping from larval occurrence to adult benthic habitat, taxa that occupy mixed or broad habitat ranges. The manuscript briefly notes transport issues earlier (**lines 50–52**) but these caveats should be revisited directly in relation to this conclusion.
Please, present this as a strong inference rather than a definitive result. Discuss hydrodynamic and ecological caveats more explicitly around **lines 267–271**.

Discussion on the points raised here have now been included in our discussion (**lines 356 – 364).** We acknowledge that our interpretation of eDNA-derived meroplankton signals required more caution. We now include discussion on how the distribution of both larvae and eDNA can be influenced by transportation, potentially decoupling detection from sites of larval origin or adult residence. We also acknowledge that larval durations vary considerably amongst taxa and that taxa may occupy more than one trait.

**7. The manuscript should distinguish more clearly between "*taxa*", "*ASVs*", and ecological diversity**
Several sections appear to move too quickly from ASV richness to ecological/taxonomic richness. For example, the manuscript states that eDNA provided "*over 4 times the number of taxa*" compared with microscopy (**lines 77–79**), but later also explains that taxonomic resolution varies by group and often remains above species level (**lines 321–328**, **399–407**).
Please, be more precise in the terminology because ASVs are not equivalent to species. Plus, increased ASV richness does not automatically imply fully resolved taxonomic richness, and "*hidden diversity*" should be qualified, especially given marker limitations.

We decided to remove the sentence *'These provided over 4 times the number of taxa'* (**line 92)** as the reviewer is correct in that taxonomic resolution varies by group in both molecular and microscope datasets. We have also changed the title to this section of our results from *'A hidden diversity of zooplankton can be detected using eDNA….'* to *'Finer zooplankton taxonomic resolution can be detected using eDNA…..'* (**line 88**).

**8. The marker limitations and taxonomic uncertainty need stronger treatment**
It does discuss marker limitations (**lines 321–328**), which is good, but this section should be sharpened because the conclusions depend on V9-based assignments. In particular, the absence of Salpida in eDNA despite the availability of database sequences (**lines 96–98**) is interesting and deserves fuller treatment as a case study in marker/sampling limitations. Please, clarify expected taxonomic resolution of the 18S V9 marker across the main groups discussed, what proportion of ASVs were confidently assigned to genus/family/order, whether some apparent mismatches between datasets could be driven by taxonomic uncertainty rather than ecological differences, and possible reasons for failure to detect Salpida. Maybe focus on these lines: **88–98**, **321–328**, **390-398**, **399-407**.

Our original text implied that all molecular methods could not replicate the species-level detail provided by a trained taxonomist. We have made an amendment in **line 393** to ensure the reader that we are discussing our 18S rRNA V9 marker data only.

There is a great deal of additional information with the supplementary tables regarding group-level taxonomic resolutions. We have added an additional sentence '*For example, Copepoda groups can be identified to Genus level with confidence, but other groups can only be identified to Family (e.g. Appendicularia, several Medusae), Order (e.g. Anthozoa and Bivalvia) or even Class (e.g. Echinodermata).* (**lines 397 - 399**) to provide a flavour of the information contained within the supplementary Tables.

We noted Salpa as they first appeared at station L4 in June and July of 2022. Since this date, very large summer blooms have occurred. We do not believe that this represents failure of the molecular method: in the summer of 2022, the Salpa were patchy in distribution and exhibit strong diurnal migration. We have added an additional statement (**line 112**) to clarify this point.

**9. Editorial revision**
There are some typographical/grammatical problems throughout the manuscript.

We thank the reviewer; all errors have been carefully corrected throughout the manuscript.

**Minor comments**

**Lines 16–18**: "*we show that nets and eDNA tallied remarkably well*" is too informal and somewhat overstated. Please, replace with a more quantitative statement.

Replaced with *produced highly consistent patterns'* (**line 17**)

**Lines 22–25**: this mechanistic conclusion is too strong for the evidence shown, please soften.

The entire abstract has been re-written to be more balanced.

**Lines 37–42**: very nice framing of the problem, but it would help to be more explicit about why trait resolution is particularly poor for meroplankton.

As an explanation, we have added a statement saying that meroplankton larvae are often identified to broad taxonomic levels by microscopy (**line 47**).

**Lines 43–54**: this section would benefit from a more balanced statement that eDNA provides different rather than uniformly better information.

Agreed: we have re-written this sentence (**line 57**) *'….eDNA-derived data provides a different perspective on plankton community structure rather than a direct replacement for microscope-based analyses. As a result, proof of concept studies that directly compare data derived from microscope and molecular methods are essential for understanding how these approaches can be combined to monitor and assess ecosystem health*.'

**Lines 55–72**: good motivation, but the last part already reads like a conclusion. Please, reduce interpretive language here.

Agreed: this has been re-written to remove results and discussion (**lines 77 – 85**).

**Lines 80–87**: the distinction between proportion of ASVs and proportion of sequences is important and should be more consistently emphasized throughout.

The sentence this refers to has been removed (see above). We have taken care throughout the rest of the manuscript to clarify differences between molecular (taxonomic presence and relative sequence abundance) and microscope-derived (direct measure of density) data.

**Lines 109–118**: please report the range of Spearman correlation coefficients in the main text, not only significance counts.

To avoid overloading the main text with detailed statistics across numerous taxa, we focused on significant correlations and their ecological interpretation in the Results. We have now added the significant Spearman correlation coefficients within **Supplementary Table 3**.

**Lines 123–131**: "trends also matched" would be stronger if accompanied by quantitative summary.

Clarified as '*monthly resolution occurrence patterns*' (**lines 143 and 152**).

**Lines 132–134**: if meroplankton annual-average trends did not match, this limitation should be discussed more explicitly.

Agreed: we have added a sentence (**line 155**): '*Given that the two approaches sample different parts of the water column and quantify different properties of the plankton community, this reduced correspondence likely reflects methodological complementarity rather than inconsistency'.*

**Lines 198–205**: please specify how chlorophyll and temperature data were aggregated and detrended.

This detail has been added to the Methods section (**line 524**): '*Annual average temperature and chlorophyll data (data (available to download from https://www.westernchannelobservatory.org.uk/) were compared to meroplankton abundance. A simple linear de-trending approach was applied prior to correlation analyses with temperature and chlorophyl to remove shared long-term trends and reduce the risk of spurious correlations'*.

**Lines 212–217**: "highly diverse, with marked seasonality" would benefit from some quantitative support in the main text.

This sentence has now been improved (**line 246**): '*Sequence data shows that polychaeta meroplankton are taxonomically diverse and exhibit marked seasonality, with different taxa peaking at different times of the year*'

**Lines 225–231**: taxon naming should be checked carefully: "Ascidiaceae," "Molgulide," and "Styledidae" likely need correction/standardization.

Care has been taken to correct these.

**Lines 253–271**: this section is interesting but currently too definitive. The absence of positive temperature correlations does not establish that increases are not temperaturerelated.

Agreed. The sentence has been changed to (**line 315**): '*For Polychaeta, de-trended annual average data showed a negative trend with temperature, indicating that the observed increases in polychaeta meroplankton are not simply or directly explained by temperature alone*.'

**Lines 272–290**: this is the conceptual core of the paper, but the interpretation needs more explicit acknowledgement that the evidence is indirect.

Agreed. The paragraph has been changed. In particular, we have modified the final sentence (**line 347**): '*Under this interpretation, the observed increases in meroplankton abundance may primarily reflect changes in water column processes rather than direct benthos responses and may represent occupancy of ecological space associated with declining suspension-feeding copepods*.'

**Lines 301–307**: well stated overall.

**Lines 308–319**: the practical monitoring implications are promising, but please avoid over-generalizing from a single site/marker/sampling design.

This has been addressed in point 8 above.

**Lines 320–353**: this section is useful and should perhaps be moved earlier in the Discussion because it provides important context for interpreting the results.

Our preference is to lead with our ecological interpretation of the data, rather than the comparison between methodological approaches.

**Lines 354–364**: strong concluding point, this section is persuasive.

**Lines 368–374**: please check "*cells per millilitre*" for zooplankton abundance, this seems unusual and may be an error in units.

Addressed; individuals $m^{-3}$ (**line 451**).

**Lines 369–370**: were the two replicate net samples averaged before analysis? Please, state explicitly.

This has been clarified (**line 445**); the values analysed represent the average of two replicate hauls.

**Lines 379–388**: helpful detail on extraction, but contamination control is not described.
**Lines 389–398**: please state whether PCR negatives, extraction blanks, or field blanks were used and how they were assessed.

More detail has now been provided on molecular methods including PCR conditions, controls used and sequencing (**lines 466 – 488**).

**Lines 399–407**: the manual taxonomic curation procedure needs clearer reproducibility criteria.

We chose to publish the entire Animalia dataset, including the taxonomic IDs provided by PR2, Silva and NCBI alongside a representative sequence for each ASV (within Supplementary Table 1). Along with the raw data deposit (BioProject ID PRJNA1045854), this allows future users of the data to scrutinise both the raw data, and to make use of our subset of Animalia sequence data. Provision of the sequence data alongside the ASV table allows the opportunity to re-analyse the data in depth as DNA barcode databases improve.

**Lines 410–417**: please describe the detrending method explicitly and define exactly which chlorophyll/temperature series were used.

This has been addressed in point 8 above.

**References**

A few references and formatting details should be checked:

**Lines 439–441**: page range appears incorrect (″805-1815″). Should it be 1805?

**Lines 529–530**: page range ″188-3201″ appears incorrect. Should be 3188?

**Lines 538–539**: ″Eco system″ should be Ecosystem?

These have been corrected.

***Reviewer #1***
The ms is clearly interesting, providing new knowledge to the seasonality and between-year variability in the plankton community. ***They speculate that the observed community changes are in relation climatic impacts linked to declining nutrient supply and a concurrent shift towards smaller size-structure in the phytoplankton community and affect the benthic-pelagic coupling***. ***This is a bit speculative, I think.*** However, using this framework to classify groups increasing in (relative) abundance as "climate winners" (or losers) both in the title and elsewhere (e.g. .22, 41, 42, etc.) is a bit misleading and not needed to identify species or groups that increase or decrease in apparent abundance between seasons and years based on a time-series analysis over a relatively short period (11 y). Judging by results presented in Fig. 3 and 4 it seems likely that what groups could be assigned to the winning and losing fractions is quite sensitive to the time periods of investigation (start the analysis two years later or end it two years earlier and you are likely to get different results). Claims about trends in the data related to climatic forcing therefore seems to me to be somewhat speculative long shot, given the relatively limited data set. ***I think the paper would benefit from an analysis of how seasonal variability and between year variability adds to the complexity and dynamics of plankton communities and would make for a very interesting, and quite possibly much cited, paper.***
I cannot validate the molecular parts of the methodology; however, the statistical analysis seems valid and appropriate and all methods should be possible to replicate (provided access to data and similar samples).

We thank the reviewer for this insightful comment and agree that assigning taxa to "winning" and "losing" categories based solely on a relatively short (11-year) molecular time-series would be overly strong and sensitive to the specific period analysed. However, our conclusions do not rely on the molecular data alone. The identification of long-term increases and decreases, and the associated trait-based patterns, is based primarily on the 35-year microscope time-series (1988–2022), which provides the statistical power to detect robust monotonic trends across seasons and years. The molecular time-series (2012–2023) is used to complement this longer record by providing enhanced taxonomic resolution, particularly for meroplankton groups that cannot be reliably resolved by microscopy.

However, we agree that the terminology "climate winners" and "climate losers" risks overstating causal inference and obscuring sensitivity to the choice of time window. We have therefore removed this terminology throughout the manuscript (see our response to Reviewer #4's comments above) and now refer explicitly to taxa showing increasing or decreasing trends over the study period. We have also moderated language linking observed patterns to climatic forcing, emphasising that any such links are indirect and interpreted within the context of the longer-term microscope record rather than inferred solely from the molecular data.

Finally, we now explicitly acknowledge that our conclusions focus on consistent trait-based patterns observable across the long-term time-series, rather than the precise classification of individual taxa.

In response to the reviewer's comments on additional analysis of seasonal variability and between year variability. We thank the reviewer for this constructive suggestion and agree

that explicitly partitioning seasonal versus interannual variability could provide additional insight into plankton community dynamics. However, such analyses would require substantial additional modelling beyond the scope of the present study, which focuses on comparing long-term trends and seasonal structure across molecular and microscope-based datasets. We have therefore retained our existing analytical framework but now clarify in the manuscript how seasonality and interannual variability are accounted for within this approach (see the response to Reviewer #4 above).

Specific comments, with recommendations for addressing each comment
1. The end of the Introduction chapter (l.66 -72) seems to me to be stating and discussing own results somewhat prematurely. I suggest moving these statements to the Results- and Discussion chapters.

Agreed: this section no longer refers to any results or discussion (**lines 77 – 85**).

2. l.164. the stratified period is defined as April – August, but in l.186. it is from May – August. Probably a simple mistake, but quite confusing when trying to interpret the results in Fig. 3, and relating to results presented in Fig. 4, where seasons and months are used instead.

Stratification occurs during May – August at Station L4. This has been amended (**line 190**).

3. In Fig. 4. I struggle to understand what criteria was used in the selection of taxonomic and/or functional groups from B) that is shown in C). Please provide more information in figure legend!

We have split Figure 4 into two: Figure 4 retains the morphological data whereas a new Figure 5 shows the molecular data. We have re-written the figure legend to make it clearer that panel A is taxonomic (focusing only on Ascidiacea, Echinodermata and Polychaeta), whereas panel B contains a mixture of taxonomic and trait aggregations. The trait-based groupings shown in panel B are not subsets of the taxonomic groups in panel A but are independent aggregations of ASVs based on inferred adult traits.

4. In Methods (l. 371-373) I find it a bit unclear why they have included biomass as it seems to contribute little to the main story, but necessitates a longer section (l. 329 – 353) that mainly deals with providing explanations for why their eDNA-approach does not match the biomass data. I find this more confusing than aiding in clarifying the main results. While it is interesting that the results deviate from other similar studies this may be an additional topic, perhaps better approached by a separate study. Thus, I do not see that the analysis benefit much from adding biomass data and recommend cutting the biomass part altogether from the presentation.

We have now included a sentence in the Methods to say why it is important to compare abundance and biomass data (**line 508**): ‘. *Comparisons were made with both abundance and biomass data because eDNA sequence data do not translate directly to organism counts and may align variably with numerical versus size-weighted (biomass) representations across taxa*’. This is highlighted in Figure 1. When discussing our findings we say ‘*Several studies have concluded that molecular-derived zooplankton data can produce similar relative abundance of taxa to those produced by morphological analyses, often with results more comparable to biomass data. Our eDNA data provided a different perspective on zooplankton composition when compared to microscope-based abundance or biomass data’* (**lines 406 - 408**).

5. Also (l.373 – 374) why report abundance values of matazoans as “cells per millilitre” instead of individuals per volume unit?

Addressed; individuals m$^{-3}$ (**line 450**).

***Reviewer #2***

**Specific comments, with recommendations for addressing each comment:**

1. The title refers to zooplankton taxa as “climatic winners and losers.” While this framing is compelling, the authors should clarify that these “winners and losers” are linked primarily to climate-driven ecosystem restructuring rather than direct temperature responses alone.

Following this reviewers’ suggestions, the title has been changed to ‘eDNA **complements microscopy to identify shared traits of zooplankton taxa that are increasing or decreasing under climate change’**

2. The authors are careful about the limitations of relative sequence abundance; however, in places the language implies stronger quantitative correspondence than is warranted (e.g. “increases” or “declines” inferred from sequence proportions). The authors are encouraged to emphasise that eDNA trends reflect relative changes in occurrence or dominance rather than absolute abundance or biomass, particularly when discussing meroplankton.

The following changes have been made to ensure that increases or decreases to trends derived from eDNA data explicitly state that this is relative abundance data.

- **Line 19:** *‘The far greater diversity and trait resolution from eDNA revealed that the meroplankton increases was driven by soft sediment (mainly sublittoral) fauna, rather than from rocky nearshore’* has been changed to *‘The far greater taxonomic insight of meroplankton groups provided by eDNA revealed a shift towards higher relative sequence abundance of taxa associated with soft sediment (mainly sublittoral) fauna, rather than from rocky nearshore’.*
- **Line 135:** ‘*Despite having only 11 data points for comparison, annual average abundance data matched for eight holoplanktonic crustacean species…*’ has been changed to ‘*Despite having only 11 data points for comparison, annual average abundance data matched in their relative temporal patterns for eight holoplanktonic crustacean species…’*
- **Line 250: ‘***Over the 11-year eDNA time-series, annual relative sequence abundance of meroplankton produced by adult suspension feeding Polychaeta have increased….’* has been changed to *‘Over the 11-year eDNA time-series, annual relative sequence abundance of meroplankton produced by adult suspension feeding Polychaeta showed an upward temporal trend in relative sequence representation….’*.
- **Line 259: ‘***Over the 11-year eDNA time-series, the relative sequence abundance of Echinoidea has significantly increased…*’ has been changed to *‘Over the 11-year eDNA time-series*, *the relative sequence abundance of Echinoidea showed a significant increasing trend through time in relative sequence contribution…’*
- **Line 263: ‘***Although Ascidiacea are rarely present in the L4 microscope dataset, the molecular data indicates Ascidiacea meroplankton to be in higher abundance*’ has been changed to ‘*Although Ascidiacea are rarely present in the L4 microscope dataset, the molecular data indicates Ascidiacea meroplankton to occur more prominently in the eDNA data….’*
- **Line 274:** *‘….the relative sequence abundance of meroplankton from soft habitats has gradually increased…*’ has been changed to *‘….the relative sequence abundance of meroplankton from soft habitats showed a significant increasing trend in relative contribution over time….*’
- **New Figure 5 legend (lines 289 – 305)** ensure explicit references to trends in eDNA data refer to relative sequence abundance as follows: ‘*Average seasonal relative sequence abundance of selected major meroplankton taxonomic groups (Ascidiacea, Echinodermata and Polychaeta). (B) Key temporal trends, including the downward trend in relative sequence abundance of Ascidiidae and the upward trend in relative*

*sequence abundance of Echinoidea alongside long-term trends based on aggregations by functional traits. For Polychaeta, ASVs were grouped according to inferred adult feeding mode (predator, deposit or suspension feeder; in red), and for all meroplankton, ASVs were grouped according to their adult life-stage habitat (hard or soft, in purple). Significant upwards or downwards monotonic trends in annual relative sequence abundance were assessed using the Mann Kendall tests, the corresponding Z value shown on the plot (n = 11)*'.

- **Line 322:** *'…our molecular time-series provided compelling evidence that increases may be from the adult species that reside in soft sediment habitats…'* has been changed to *'…our molecular time-series provided compelling evidence that the relative contribution of meroplankton adult species that reside in soft sediment habitats rather than on hard substrata has increased…'*
- **Line 337**: *'Likewise, for meroplankton, the broad range of taxa detected with sediment-dwelling adult stages included…..'* has been changed to '*A similar patterns is evident for meroplankton, where the taxa showing increasing relative sequence representation included a broad range of taxa detected with sediment-dwelling adult stages…'*

3. The conclusion that increasing meroplankton originates primarily from soft-sediment adult habitats is important. However, processes such as transport, larval duration, and hydrodynamics could also influence these patterns. The authors should acknowledge alternative explanations and clarify why the adult habitat signal is considered robust.

This point was also raised by Reviewer #4 (comment no. 6) and has been addressed.

4. Please clarify whether there was a specific reason for not using COI mini-barcode primers, given that COI reference databases for zooplankton are generally more extensive than those for 18S.

We selected the 18S rRNA V9 marker because it provides broad and relatively unbiased amplification across metazoan zooplankton, including gelatinous taxa and meroplanktonic larvae that are central to this study. COI metabarcoding is known to suffer from primer bias and inconsistent COI amplification: COI primers are taxonomically biased and known to fail or underperform for some Copepoda groups, Appendicularia, Thaliacea and many Cnidaria), which would have reduced taxonomic coverage and comparability with the morphological time-series. The 18S rRNA V9 region has also previously been used to study zooplankton communities (see Bucklin et al., 2019; Albaina et al., 2016; Lindeque et al., 2013). In addition, as our objective was to assess relative temporal patterns and functional restructuring across the zooplankton community rather than to maximise species discrimination, we selected the 18S V9 marker. We now clarify this rationale in the Discussion (**lines 397**) and the Methods (**lines 468 - 473**).

5. The absence of Salpida in the eDNA dataset despite their presence in net samples is noted but not explored in depth. Given their ecological importance and relevance to climate change, this warrants further discussion. The authors are encouraged to expand on possible explanations, including primer bias, vertical distribution, and marker limitations.

This point has been addressed above (see Reviewer 4, point no. 8).

6. General comment regarding colour usage in figures & tables: The colours orange and blue are used for multiple figures and tables, but in different contexts, which can be confusing, e.g. in Figure 2 & Suppl. Tables 2 & 3: blue = morphological data, orange = eDNA data; but in Figures 3 & 4: blue = downward trend, orange = upward trend.
Also, in Figure 4a, there is no separate legend to explain the gradation in blue shading used for the monthly data in the left-hand section of the figure, so does the legend on the far right apply to the monthly data shown in Fig 4a, or just the section to the right (seasonal trend)? It

is thus unclear if the shades of blue indicate relative monthly abundance, or the extent of downward trend for each month over the time series, or both. This needs clarification. Consider using different colour pairs to distinguish between eDNA/morphological data and downward/upward trends (maybe red or magenta vs green?). Please check the colours as used in Figure 4a and explain what the monthly shaded squares represent in the legend.

Thank you for pointing this out. The colours in **Figure 2** have been changed (now grey squares for microscope data and red triangles for eDNA). We avoid confusion, we have split **Figure 4** into two. **Figure 4** now displays only microscope data with two clear panels: the first showing seasonal patterns in abundance (now in red with a new legend) and the second showing trends. A new **Figure 5** focuses solely on eDNA data. The colours have also been changed in **Supplementary Table 3**: eDNA relative abundance data is now displayed in red and microscope data in grey.

7. The Methods outlined are inadequate or inappropriate in two places. (a) Lines 371-374: these methods and units (cells per ml) are surely relevant for "diatoms, dinoflagellates and other protists" according to the reference cited - not zooplankton. Please correct as appropriate for zooplankton, with the relevant reference/s. (b) Lines 390–392: Information regarding PCR reagents and cycling conditions is missing. For reproducibility, it is recommended that all PCR conditions and reagent details be explicitly included.

Following similar requests from Reviewers 1 and 4 above, the methods section has now been re-written and contains the additional detail requested.

**Minor comments:**

1. Terms such as "morphology," "microscope data," and "traditional methods" are used interchangeably. The authors are encouraged to standardise terminology throughout the manuscript.

Agreed. All references to morphology have been removed and traditional methods reduced substantially. We have chosen to use the term microscope/microscopy as it more accurately describes what the data are.

2. Some Mann–Kendall results are described in the text without always specifying sample size or time window; the authors are encouraged to ensure all reported statistics clearly indicate the dataset and temporal scope used.

We have taken clearer to ensure it is clearer what data was used for each Mann-Kendall test (e.g. **line 186**, **line 218, line 253, line 283**) and the temporal scope (e.g. **line 234, line 254, line 304**).

3. Figures are information-rich but dense. The authors are encouraged to simplify legends if possible.

Figure 4 has been split into two. Figure 4 now displays only microscope data and Figure 5 focuses solely on eDNA data. The legends for Figure 3 (**lines 212 – 226**), Figure 4 (**lines 280 – 285)** and Figure 5 (**lines 295 – 305)** have been re-written to more easily interpret the figures.

4. Dates inconsistency: Results (line 77) indicate eDNA samples were collected between 2012–2022, whereas Methods (line 379) state 2012–2023. Please check and correct dates at lines 77, 379, 410, 160, 185, 198, and 241 for consistency.

eDNA data collection spanned Feb 2012 to March 2023 (**line 91, line 456**). This entire dataset was used when comparing monthly temporal data. For annual average data, the eDNA time-series spanned 2012 – 2022 (**line 134, line 520)**.

5. The number of samples used in different analyses is unclear. For example: Line 16: "425 paired samples over 11 years"; Line 62: "over 400 concurrently collected samples"; Lines 379–380: "415 dates"; Line 410: "415 dates"; Lines 308: "415 parallel samples analysed". Please explicitly state the number of samples used for each method (molecular, microscopy) and clarify which samples were included in combined analyses.

There were 415 matching samples (changed in **line 16** and **line 70**).

6. The taxa named in the text (lines 117-118; 127-129) as those that exhibit significant month-year or annual correlations between microscope and eDNA data do not always match what is shown in the Supplementary data – please see the detailed list of comments provided and correct accordingly.

These have been thoroughly checked, and the text now matches the Supplementary Table.

7. The crustacean taxa named in the text (lines 168-171) as having declined, regardless of whether during stratified or non-stratified seasons, does not wholly match the data depicted in Figure 3a (incorrectly cited as Figure 4b). For example, *Clausocalanus* spp. (although listed) showed no significant decline for either season, while *Ctenocalanus vanus* (not listed) showed significant declines in both seasons. *Acartia clausii* (not listed) and *Calanus helgolandicus* (listed) showed significant declines in one season only. Please revisit this and correct as intended.

These have been thoroughly checked, and the text now matches the revised **Supplementary Table 3**.

8. Lines 191-193: I would think this section should focus on the significant trends only, as shown in either blue or orange. Hence, L191 should presumably read "Plots in B and C highlight the significant increase over time for fine filter feeders during stratification". Next, L192 states "(increases…for) carnivores/detritivores when the water column is not stratified" - however, the time series are shown in grey for both stratified and non-stratified seasons, indicating no significant trends. Finally, I presume L192-193 should read "and significant declines to suspension feeders/omnivores over the entire year". Please revisit and reword accordingly.

The figure legend has now been corrected (**lines 212 – 226**).

10. Lines 390–391: Please clarify whether the primers listed are the exact primers used or whether Illumina adaptor sequences were added.

More detail of the PCR cycling conditions and library preparation method are now included (**lines 466 – 488**). The method used by Nu-OMICS followed published methods by the Earth Microbiome Project.

11. A few sentences in the discussion (especially paragraphs on feeding modes and trait interpretation) could be split for readability, but this is stylistic, not required.

The discussion has been re-worked to help it flow better, this included breaking paragraphs and introducing some new sentences.

**Detailed list of typographical, formatting, grammatical or other issues:**

All listed typographical and grammatical errors have been corrected.

The reviewer noticed that in Supplementary Table 1, the ID assigned in this study does not always match the ID given by PR2. Discrepancies between PR2, SILVA and NCBI taxonomic assignments are not uncommon and often reflect database versioning, taxonomic updates, or legacy annotations. In this study, final taxonomic assignments were therefore

determined following manual inspection of ASV sequences, sequence alignment, and cross-comparison with the most up-to-date reference databases to ensure consistency and biological plausibility.

Response to comments:

| | | |
|---|---|---|
| Title | Editor suggested manuscript title ("increasing or decreasing" in current version is confusing and should be revised): eDNA and microscopy reveal traits underlying long term zooplankton community change | Suggestion accepted; title has been amended (lines 1 – 2) |
| Display items | Editorial request: currently, the resolution of text elements in some figures appears to be poor/grainy (e.g., Figures 4 & 5); please ensure the text elements within the figures are rendered at high resolution. | Higher resolution versions of Figures 4 and 5 have been uploaded. |
| Source Data | All source data, i.e. the numerical results underlying the graphs and charts presented in the main figures,are mandatory and must be made available as Supplementary Data (in Excel or text format) or via a generalist repository (e.g., Figshare or Dryad). | The data availability statement was included, but perhaps not in the correct place. It is now at the end of the manuscript (lines 581 – 588). All data used within the study are fully accessible. |
| Source Data | Please include a caption for the source data file and add a sentence to the Data Availability statement to describe how source data can be accessed using text similar to "The source data for Figure X is in Supplementary Data Y". | This has been included in the Data Availability Statement (see lines 583 and 587). |
| Data | Authors should cite any datasets stored in external repositories that are mentioned within their manuscript. For previously published datasets, we ask authors to cite both the related research articles and the datasets themselves. For more information on how to cite datasets in submitted manuscripts, please see our submission guidelines. | The microscope zooplankton time-series data are available via the British Oceanographic Data Centre (BODC) (line 582). The citation for this dataset is included within the references (no. 19) (line 476). Raw sequence reads have been deposited in the NCBI Sequence Read Archive under BioProject ID PRJNA1045854 (line 585). |
| Press/Social Media | We regularly highlight papers published on the journal's Social Media accounts. If you would like us to mention authors, institutions, or lab groups in these posts, please provide the social media along with the respective handles in the right-hand column. Please | PML's communication team are preparing social media posts related to this communication.<br>LinkedIn: Plymouth Marine Laboratory (PML)<br>X/Twitter: @PlymouthMarine<br>BlueSky: @pml.ac.uk |

| | | |
|---|---|---|
| | let us know if there are any plans for an Institutional press release on your article. | |
| Figure Captions | Please remove figures from the article file and ensure that all main text Figures 1-5 captions are grouped together at the end of your main manuscript file. Embedding captions within figure files is not permitted at this stage. | Done; Figure legends are now located at lines 590 – 634. |
| Titles and Captions | GUIDANCE: Figure and Table Captions: Please ensure that all Main Text Figures and Tables include both titles and captions. The titles must describe the figure as a whole and must not contain reference to specific figure panels. The titles should be brief. All figure panels must be defined in the legend. Figure legends should be informative, clear, not unnecessarily long. | Figure legends have been re-written (lines 590 – 634):<br>• Shorter, informative titles<br>• Figure 2 panels now defined<br>• Greater clarity, with information removed to methods where possible (see Figure 1 legend) |
| Citations | We note thatFigure 2a and 4c panels are cited in the article file but no such panels are present in he figure files itself; Please correct this upon resubmission. | Corrected. |
| Supplementary Information | Supplementary Information must be provided as a single, separate file in PDF format. Please combine supplementary Tables 2,3,4 into a single file and provide as supplementary Information PDF. | Completed: Combined as new Supplementary Tables 1, 2 and 3. |
| Main Text | GUIDANCE: Please check that you have not copied any text directly from published work (even your own) without clear attribution, including one or more references. We run a plagiarism detection software and may need to request additional changes if we identify large blocks of identical text. | Done. |
| Author Information | Please ensure that each affiliation contains the institution name, city, and country. | Done; country added to address. |
| Data Availability | Guidance: The Data availability statement should include:<br>- The data types used in the study and how they can | Please see our data availability statement (lines 581 – 588). |

| | | |
|---|---|---|
| | be accessed<br>- Accession codes for deposited data<br>- Other unique identifiers (such as DOIs and hyperlinks for any other datasets)<br>- If applicable, a statement explaining any restrictions on data access<br>- If data are reported in the study, the statement should specify, at a minimum, that all relevant data are available from the authors upon request. This statement should include details on who will be responsible for replying to this request.<br>- If no data were used or generated in the study, the Data Availability statement should be as follows: 'Data sharing not applicable to this article as no datasets were generated or analysed during the current study.'<br><br>You should also state how the data are currently being stored. If a dataset has a Digital Object Identifier (DOI) as its unique identifier, we ask that you include this in the Reference list and cite the dataset in the Data Availability<br>Statement.<br><br>For data restriction (on generated data): We require the following parameters in DAS-reasons for controlled access, precise conditions of access (including contact details for access requests), a timeframe for response to requests and details of any restrictions imposed on use of controlled data by reporting conditions of data use agreements.<br><br>For manuscripts that contain substantial microscopy image data - We strongly encourage the deposition of | *All data used within this study are fully accessible. The microscope zooplankton time-series data are available via the British Oceanographic Data Centre (BODC; see19 to download). This data was used to generated Figures 1a, 1b, 2, 3 and 4), Raw sequence data have been deposited in the NCBI Sequence Read Archive under BioProject ID PRJNA1045854. In addition, details of Animalia ASVs identified in this study, including a representative sequence, taxonomic classification together with the ASV table are within Supplementary Data. This Animalia-specific ASV table was used to generate Figures 1, 2 and 5).* |

| | | |
|---|---|---|
| | full microscopy image data sets in the Image Data Resource (https://idr.openmicroscopy.org/about/). Mandatory deposition is required for:<br>● Protein sequences<br>● DNA and RNA sequences and sequencing data<br>● Genetic polymorphisms<br>● Linked genotype and phenotype data<br>● GWAS summary statistics<br>● Macromolecular structure<br>● Microarray data (must be MIAME compliant)<br>● Mass spectrometry-based proteomics data<br>Small molecule crystallographic data must be deposited with the CCDC or the ICSD. Accession codes must be provided in the "Data Availability" section. Please provide sepcific link for microscope zooplankton time-series data used from British Oceanographic Data Centre BODC database. | |
| Competing Interests | We note that Competing Interest statement is missing from the article file. Please ensure that your 'Competing interests' statement accounts for both financial and non-financial interests. All authors must be accounted for within the statement, e.g. with use of 'all other authors declare no competing interest' where relevant. If any updates are made to your manuscript's 'Competing Interests' statement, please also update the manuscript tracking system with the text of the new statement.<br><br>For patents, full details including patent name, number, authors, and geographical region should be provided. If no competing interests exist, the statement 'The authors declare no competing interests' should be given. See our competing interests policy for further information: | Now included (lines 578 – 579).<br>The authors declare no competing interests. |

| | | |
|---|---|---|
| | https://www.nature.com/nature-research/editorial-policies/competing-interests | |
| Acknowledgements | Any relevant funding should be declared in a separate funding statement. Please refer to our funding statement guidelines for more information. https://www.nature.com/nature-portfolio/editorial-policies/funding<br>After making any changes, please update the manuscript tracking system with the text of your new acknowledgements statement. | This was included, but possibly in the wrong location.<br>Please see lines 561 – 569. |
| Primers/oligonucleotides | Sequences of oligonucleotides (e.g. primers, RNAi, Crispr), or company names and catalog numbers if reagents are commercial, should be provided in the Methods. If this information is lengthy, it may be provided in Excel format, as separate Supplementary Data, mentioning the file in the Methods. | Alread included: please see lines 362 and 363.<br>*The 18S rRNA V9 region was amplified with primers Euk_1391F (GTACACACCGCCCGTC)[41] and EukBr (TGATCCTTCTGCAGGTTCACCTAC)[42].* |
| Statistics | Please indicate the statistical test used for data analysis and where appropriate, please specify whether it was one-sided or two-sided and whether adjustments were made for multiple comparisons, in the legend(s) of figure 1C. | Figure 1 legend has been amended to include this information (line 595). |
| Figure Quality | Each complete figure must be supplied at 300 dpi or higher resolution to ensure the highest-quality reproduction in the journal. Please submit your figures following the guidelines: https://www.nature.com/commsbio/submit/submission-guidelines#figures-publication | Higher quality Figures have been provided. |
| Supplementary Data | Please label supplementary table 1 as supplementary data and change citations accoringly in the article file. Please provide description of supplementary data. Pleas re-label supplementary tables accordingly, ensure to change citations in the article file. | Supplementary Table 1 is now changed to Supplementary Data, with all additional Supplementary Tables re-labelled accordingly throughout the document. |

**Reviewer Comments – Communications Biology**

**Title:** eDNA complements microscopy to identify shared traits of zooplankton winners and losers under climatic warming

**Authors:** Karen Tait, Penelope Lindeque, Amanda Beesley, Andrea McEvoy, Helen Parry, and Angus Atkinson

**Brief summary of the manuscript:**

This manuscript compares long-term zooplankton data obtained from traditional morphological identification of samples collected using 200 µm WP2 nets against an 11-year eDNA time series data (2012 – 2023) at Station L4 in the Western English Channel. Using both morphological and molecular approaches, this manuscript aimed to examine taxonomic trends and functional traits of zooplankton "winners and losers" under climatic warming.

The manuscript displayed strong agreement between both methods for dominant holoplankton taxa, while eDNA revealed higher taxonomic resolution for meroplankton. The results from this study suggest that increases in meroplankton are primarily associated with fine filter-feeding taxa originating from soft-sediment habitats, which are likely linked to indirect climate effects mediated through stratification, nutrient supply, and phytoplankton size structure rather than through temperature alone.

The manuscript is suitable for publication after minor revision (possibly this qualifies as major revision given the extent of minor edits required?)

**Overall impression of the work:**

This is a strong, timely, and carefully executed study and is highly significant to plankton ecology, marine food web dynamics, and climate change research. The manuscript clearly demonstrates that eDNA complements, rather than replaces, traditional monitoring based on microscope identification of zooplankton, and strengthens the argument for molecular-based approaches to monitoring biodiversity.

The manuscript is generally well-written, and the statistical analyses seem appropriate. However, the manuscript contains a substantial number of minor errors (e.g. taxonomic misspellings, incorrect Figure or Table referencing, mismatch between text and data), which suggests the final checking was rushed.

**Specific comments, with recommendations for addressing each comment:**

1. The title refers to zooplankton taxa as "climatic winners and losers." While this framing is compelling, the authors should clarify that these "winners and losers" are linked primarily to climate-driven ecosystem restructuring rather than direct temperature responses alone.

2. The authors are careful about the limitations of relative sequence abundance; however, in places the language implies stronger quantitative correspondence than is warranted (e.g. “increases” or “declines” inferred from sequence proportions). The authors are encouraged to emphasise that eDNA trends reflect relative changes in occurrence or dominance rather than absolute abundance or biomass, particularly when discussing meroplankton.

3. The conclusion that increasing meroplankton originates primarily from soft-sediment adult habitats is important. However, processes such as transport, larval duration, and hydrodynamics could also influence these patterns. The authors should acknowledge alternative explanations and clarify why the adult habitat signal is considered robust.

4. Please clarify whether there was a specific reason for not using COI mini-barcode primers, given that COI reference databases for zooplankton are generally more extensive than those for 18S.

5. The absence of Salpida in the eDNA dataset despite their presence in net samples is noted but not explored in depth. Given their ecological importance and relevance to climate change, this warrants further discussion. The authors are encouraged to expand on possible explanations, including primer bias, vertical distribution, and marker limitations.

6. General comment regarding colour usage in figures & tables: The colours orange and blue are used for multiple figures and tables, but in different contexts, which can be confusing, e.g. in Figure 2 & Suppl. Tables 2 & 3: blue = morphological data, orange = eDNA data; but in Figures 3 & 4: blue = downward trend, orange = upward trend.

Also, in Figure 4a, there is no separate legend to explain the gradation in blue shading used for the monthly data in the left-hand section of the figure, so does the legend on the far right apply to the monthly data shown in Fig 4a, or just the section to the right (seasonal trend)? It is thus unclear if the shades of blue indicate relative monthly abundance, or the extent of downward trend for each month over the time series, or both. This needs clarification.

Consider using different colour pairs to distinguish between eDNA/morphological data and downward/upward trends (maybe red or magenta vs green?). Please check the colours as used in Figure 4a and explain what the monthly shaded squares represent in the legend.

7. The Methods outlined are inadequate or inappropriate in two places. (a) Lines 371-374: these methods and units (cells per ml) are surely relevant for “diatoms, dinoflagellates and other protists” according to the reference cited - not zooplankton. Please correct as appropriate for zooplankton, with the relevant reference/s. (b) Lines 390–392: Information regarding PCR reagents and cycling conditions is

missing. For reproducibility, it is recommended that all PCR conditions and reagent details be explicitly included.

**Minor comments:**

1. Terms such as “morphology,” “microscope data,” and “traditional methods” are used interchangeably. The authors are encouraged to standardise terminology throughout the manuscript.

2. Some Mann–Kendall results are described in the text without always specifying sample size or time window; the authors are encouraged to ensure all reported statistics clearly indicate the dataset and temporal scope used.

3. Figures are information-rich but dense. The authors are encouraged to simplify legends if possible.

4. Dates inconsistency: Results (line 77) indicate eDNA samples were collected between 2012–2022, whereas Methods (line 379) state 2012–2023. Please check and correct dates at lines 77, 379, 410, 160, 185, 198, and 241 for consistency.

5. The number of samples used in different analyses is unclear. For example: Line 16: “425 paired samples over 11 years”; Line 62: “over 400 concurrently collected samples”; Lines 379–380: “415 dates”; Line 410: “415 dates”; Lines 308: “415 parallel samples analysed”. Please explicitly state the number of samples used for each method (molecular, microscopy) and clarify which samples were included in combined analyses.

6. The taxa named in the text (lines 117-118; 127-129) as those that exhibit significant month-year or annual correlations between microscope and eDNA data do not always match what is shown in the Supplementary data – please see the detailed list of comments provided and correct accordingly.

7. The crustacean taxa named in the text (lines 168-171) as having declined, regardless of whether during stratified or non-stratified seasons, does not wholly match the data depicted in Figure 3a (incorrectly cited as Figure 4b). For example, *Clausocalanus* spp. (although listed) showed no significant decline for either season, while *Ctenocalanus vanus* (not listed) showed significant declines in both seasons. *Acartia clausii* (not listed) and *Calanus helgolandicus* (listed) showed significant declines in one season only. Please revisit this and correct as intended.

8. Lines 191-193: I would think this section should focus on the significant trends only, as shown in either blue or orange. Hence, L191 should presumably read “Plots in B and C highlight the <u>significant</u> increase over time for fine filter feeders during stratification”. Next, L192 states “(increases…for) carnivores/detritivores when the water column is not stratified” - however, the time series are shown in grey for both stratified and non-stratified seasons, indicating no significant trends. Finally, I

presume L192-193 should read “and significant declines to suspension feeders/omnivores over the entire year”. Please revisit and reword accordingly.

10. Lines 390–391: Please clarify whether the primers listed are the exact primers used or whether Illumina adaptor sequences were added.

11. A few sentences in the discussion (especially paragraphs on feeding modes and trait interpretation) could be split for readability, but this is stylistic, not required.

**Detailed list of typographical, formatting, grammatical or other issues:**

Introduction:

- L34: presumably replacement by, not of, more carnivorous forms?
- L35: “A variety of mechanisms have been proposed. These included…”

Results:

- L79: missing a fullstop
- L104: Platyhelminth**e**s
- L117: *Acartia*, not *Arcatia*
- L117-118: text does not fully match the data shown - according to the significant correlations shown in Suppl. Table 3, you should remove *Centropages* and add *Podon*
- L118: Supp Table 3 (not 4)
- L118: You cite Figure 2a (but not 2b later on), and the Figure legend only has upper panel and lower panel, not a and b
- L127: text does not fully match the data shown - according to the significant correlations shown in Suppl. Table 3, you should remove *Aglantha*
- L128: Supp Table 3 (not 4)
- L129: to be consistent, according to the significant correlations shown in Suppl. Table 3, you should add *Lizzia* and *Solmaris*
- L129: Figure 2 (not 3)
- L160 (and elsewhere): the Mann Kendall test (or Mann-Kendall test?) Perhaps expand to say “the Mann-Kendall test for monotonic trend”
- L166-167: Figure 3 (not Figure 1 - in 3 places)
- L172: I think Figure 4b should be Figure 3a?
- L185: Table 4 (not 1)
- L186: for each taxon (not taxa)
- L187: “stratification (May – August)” – elsewhere you have April to August
- L190: as **a** key group
- L236: correct the spelling of Phoronidae, Platyhelminthes

Discussion:

- L288: and that they were filling
- L298: **con**ceivable, not perceivable
- L302: ecosystem**s**
- L325: multiple ma**r**ker

Methods:

- L370: sentence incomplete – following what?
- L371-374: these methods and units (cells per ml) are surely intended for phytoplankton - "diatoms, dinoflagellates and other protists" according to the reference cited - not zooplankton. Please correct as appropriate for zooplankton, with the correct reference/s.
- L414: tests were used
- L415: data is repeated

Suppl Dataset 1:

- Cell 27A: correct the spelling of column, and correct U-AA to U-**X**
- Row 87: the PR2 ID (*Phoronis*) does not correspond with the NCBI Scientific name or WoRMS Aphia ID (Mollusca – Polyplacophora)

Suppl Table 2:

- The black lettering on the darkest shade of blue is difficult to read
- As no biomass data **are** available
- Correct the spelling of biomass (3s's) under Microscope data

Suppl Table 3:

- Caption: -delete "and" from "(for microscope-derived data and)"
- Caption: -correct spelling of "Pearson"
- Caption says "nm" means no match, but nm is not used – it is either "n.mol" or "n.mor"; you should also explain "nd"
- Caption should explain which months the numbers 1-12 represent
- B: Chaetognath**a**

Suppl Table 4:

- Chaetognath**a**
- Bryozoan reference: "Contribution àa l'étude" – remove extra "a"
- For a number of the other references – Genus & species names should be in italics

Figure 1:

- L139: fix spelling of correlations
- L140: fix the grammar (delete "show matches")
- L145: Chaetognath**a**

Figure 2:

- Correct Y-axis spelling of propo**r**tion and ar**c**sine
- Plot titles - Genera should be in italics

Figure 3:

- 3a – correct spelling of Chaetognath**a**, *Acartia (Acartiura) clausi**i**,* check for fullstop after spp.

Figure 4:

- 4a – a legend is needed to explain the blue squares – do they indicate relative monthly abundance, or trend direction, or both? This should be properly explained in the figure caption.
- 4a – correct the spelling of: Polychaet**a,** Gastropod**a**

**Manuscript#:** COMMSBIO-25-12027-T

This manuscript combines a long-term microscopy dataset from Station L4 with an 11-year parallel eDNA time series to assess whether these two approaches converge on similar temporal patterns in zooplankton and whether eDNA can reveal the traits of climatic "winners" and "losers" with greater resolution. The paper has clear strengths: the dataset is valuable, the ecological question is really good, and the microscopy–eDNA comparison is potentially important for long-term monitoring. The most compelling aspect is the added taxonomic resolution eDNA provides for meroplankton. However, the main issues are that several conclusions are stated too strongly for the analyses presented, the statistical framework is not described clearly enough, the trait-based interpretation needs better justification and add clearer caveats around trait assignment and adult-habitat inference, and the manuscript contains numerous inconsistencies in wording, taxonomy, and language (see minor comments below).

**Major comments**

**1. The causal interpretation is stronger than the evidence currently supports**

The manuscript repeatedly moves from observed pattern description to mechanistic conclusion. For example, the Abstract states that "*the climate winners were fine filter feeders*" and that this suggests "*water column processes, mediated by declining nutrient supply and smaller phytoplankton, were driving a fundamental shift in the pelagic food web*" (**lines 22–25**). Similar language appears in the Introduction (**lines 66–72**) and Discussion (**lines 272–290**).

The data presented appear to support temporal changes in taxonomic groups, agreement between methods for some taxa, trait-associated differences in long-term trends, lack of simple positive temperature relationships for meroplankton, increasing eDNA signal from some taxa associated with soft-bottom adult habitats. However, these results do not by themselves demonstrate that reduced nutrient supply and smaller phytoplankton are the direct drivers, nor do they definitively show that meroplankton increases are driven by water column processes rather than benthic changes.

Please, moderate the causal language throughout the manuscript. Phrasing such as "*consistent with,*" "*supports the hypothesis that,*" or "*suggests an indirect pathway via food-web restructuring*" would be more appropriate unless further analyses are added.

**2. The comparison between microscopy and eDNA needs more careful framing**

A key issue is that the two methods are not directly equivalent. The microscopy data come from full-depth vertical hauls (0–50 m) using 200 μm WP2 nets (**lines 368–370**), whereas the eDNA data come from weekly surface seawater samples (5 L) (**lines 379–381**). In addition, the outputs differ, microscopy provides abundance/biomass estimates, while eDNA provides sequence-based compositional data (**lines 411–413**).

The manuscript acknowledges some of this, especially in the Discussion (**lines 334–345**), but not strongly enough in the framing of the Results and Abstract. Statements such as nets and eDNA "*tallied remarkably well*" (**lines 17–18**) or that surface eDNA sampling "*can capture the key trends*" (**lines 120–122**) should be made more cautiously and quantitatively.

Please, make this limitation much more explicit earlier in the manuscript, especially in the Abstract and Results. It should be clear that the methods sample different parts of the water column, they quantify different things, lack of agreement is not necessarily methodological failure, agreement is notable precisely because the methods are not equivalent. This will make the conclusions more credible.

**3. The statistical framework needs many more details**

The analytical methods are currently under described relative to the weight placed on the conclusions. Important details are missing or not very clear yet.

Examples:

- monthly averages are used for some comparisons (**lines 109–113**), whereas annual averages are used elsewhere (**lines 116–120**, **160–164**, **198–205**). The reason is not fully explained.
- it is unclear whether temporal autocorrelation and seasonality were accounted for in the correlation analyses.
- there is no mention of correction for multiple testing despite many correlations and trend tests.
- the de-trending procedure used before testing temperature/chlorophyll relationships is not described (**lines 203–205**, **243–244**, **415–417**).
- the manuscript defines stratified periods inconsistently: "*April–August*" (**line 164**) versus "*May–August*" (**line 186**).
- it is not clear exactly what unit was entered into each Mann–Kendall test: annual averages? Seasonal annual averages? Proportions?

- in figure 1B (**line 136**), why the biomass doesn't reach 1 if it is relative contributions, like abundance and eDNA do?

Please, improve the statistical section (**lines 409–419**) to include exact temporal aggregation used for each analysis, sample sizes for each test, how seasonality and autocorrelation were handled, how de-trending was performed, whether multiple-testing correction was considered. Why Spearman, Pearson, and Mann–Kendall were chosen for specific comparisons? How stratified vs non-stratified periods were defined and why?

**4. Trait assignments are important, but the framework is not transparent enough**

The main conclusion is that changes are linked to feeding mode, especially the distinction between fine filter feeders, suspension feeders/omnivores, and carnivores/detritivores (**lines 159–178**, **182–194**, **272–285**, **374–376**). However, the basis for assigning taxa to these categories is not sufficiently explained in the main text. Similarly, meroplankton are grouped by adult habitat (hard/soft/both) and by adult feeding mode (**lines 213–217**, **232–249**, **418–419**), but these assignments are ecologically complex and likely uncertain for some taxa.

Please, clarify whether feeding traits refer to larval stages, adult stages, or generalized taxon-level traits, how ambiguous taxa were handled, whether taxa with mixed ecology were excluded or forced into a single class, how robust the hard/soft substratum assignments are. This is important to the interpretation. I strongly suggest either adding a good explanation in the main text or adding a sensitivity analysis showing whether the key conclusions remain if ambiguous taxa are excluded. Important sections include **lines 66–72**, **159–178**, **213–217**, **232–249, and 374–376**.

**5. The "*no evidence for temperature-driven meroplankton increase*"**

The manuscript states in the Abstract that "*There was also no evidence that increases to meroplankton groups were driven by temperature*" (**lines 21–22**). Similar statements appear in the Results (**lines 201–205**) and Discussion (**lines 259–266**). This conclusion seems too strong. The analyses described appear to show only that de-trended annual average abundance was not positively correlated with temperature, and that polychaetes showed a negative relationship. That does not rule out nonlinear responses, seasonal effects, lagged responses, indirect temperature effects via stratification or food-web structure, taxon-specific responses hidden by aggregation.

Please, revise these claims to reflect what was actually tested. A more defensible wording would be something like "*We found no simple positive relationship between de-trended annual meroplankton abundance and temperature.*" Think about it!

**6. The statement that increasing meroplankton derives mainly from soft-sediment taxa is interesting, but still inferential**

One of the most interesting contributions of the study is the suggestion that the increase in meroplankton is driven by taxa whose adults inhabit soft sediments rather than rocky nearshore habitats (Abstract **lines 19–21**; Results **lines 232–237**; Discussion **lines 267–271**). This is plausible and potentially important. However, the manuscript currently presents this with too much certainty. Potential complications include advection of larvae and eDNA, transport of DNA in the water column, uncertain mapping from larval occurrence to adult benthic habitat, taxa that occupy mixed or broad habitat ranges.

The manuscript briefly notes transport issues earlier (**lines 50–52**) but these caveats should be revisited directly in relation to this conclusion.

Please, present this as a strong inference rather than a definitive result. Discuss hydrodynamic and ecological caveats more explicitly around **lines 267–271**.

**7. The manuscript should distinguish more clearly between "*taxa*", "*ASVs*", and ecological diversity**

Several sections appear to move too quickly from ASV richness to ecological/taxonomic richness. For example, the manuscript states that eDNA provided "*over 4 times the number of taxa*" compared with microscopy (**lines 77–79**), but later also explains that taxonomic resolution varies by group and often remains above species level (**lines 321–328**, **399–407**).

Please, be more precise in the terminology because ASVs are not equivalent to species. Plus, increased ASV richness does not automatically imply fully resolved taxonomic richness, and "*hidden diversity*" should be qualified, especially given marker limitations.

**8. The marker limitations and taxonomic uncertainty need stronger treatment**

It does discuss marker limitations (**lines 321–328**), which is good, but this section should be sharpened because the conclusions depend on V9-based assignments. In particular, the absence of Salpida in eDNA despite the availability of database sequences (**lines 96–98**) is interesting and deserves fuller treatment as a case study in marker/sampling limitations.

Please, clarify expected taxonomic resolution of the 18S V9 marker across the main groups discussed, what proportion of ASVs were confidently assigned to genus/family/order, whether some apparent mismatches between datasets could be driven by taxonomic uncertainty rather than ecological differences, and possible reasons for failure to detect Salpida. Maybe focus on these lines: **88–98**, **321–328**, **390-398**, **399-407**.

**9. Editorial revision**

There are some typographical/grammatical problems throughout the manuscript. Examples:

- "*gathering in pace*" (**line 44**), or should be "*gathering pace*"? Please, check it.
- "*correlatiosn*" (**line 139**) correct to "*correlations*"
- "Ascidiaceae" vs "Ascidiacea" vs "Ascidiidae" used inconsistently (**lines 86**, **225–230**, **244–245**)
- "Phonorida" vs "Phoronida" (**lines 133**, **236**)
- "*use eDNA to monitor a diversity of ecosystem*" (**lines 301–302**) should be plural "ecosystems", no?
- "multiple maker approach" (line **325**) should be "multiple marker approach". "r" is missing.

There also appear to be inconsistencies in figure numbering and cross-referencing:

- **lines 165–167** refer to "Figure 1b" and "Figure 1a" in a way that seems likely out of order..
- **lines 350–351** refer to "Figure 3" where Figure 2 may be intended ?
- **lines 230**, **235**, **245** seem to cross-reference "Figure 3b/3c" and "Figure 4b/4c" inconsistently. Please, check it.

Please, take full revision including line-by-line proofreading, checking figure numbering and references, checking taxonomic names, checking terminology across Abstract, Results, Figures, and Supplementary Tables.

**Minor comments**

**Lines 16–18**: "*we show that nets and eDNA tallied remarkably well*" is too informal and somewhat overstated. Please, replace with a more quantitative statement.

**Lines 22–25**: this mechanistic conclusion is too strong for the evidence shown, please soften.

**Lines 37–42**: very nice framing of the problem, but it would help to be more explicit about why trait resolution is particularly poor for meroplankton.

**Lines 43–54**: this section would benefit from a more balanced statement that eDNA provides different rather than uniformly better information.

**Lines 55–72**: good motivation, but the last part already reads like a conclusion. Please, reduce interpretive language here.

**Lines 80–87**: the distinction between proportion of ASVs and proportion of sequences is important and should be more consistently emphasized throughout.

**Lines 109–118**: please report the range of Spearman correlation coefficients in the main text, not only significance counts.

**Lines 123–131**: "trends also matched" would be stronger if accompanied by quantitative summary.

**Lines 132–134**: if meroplankton annual-average trends did not match, this limitation should be discussed more explicitly.

**Lines 198–205**: please specify how chlorophyll and temperature data were aggregated and detrended.

**Lines 212–217**: "highly diverse, with marked seasonality" would benefit from some quantitative support in the main text.

**Lines 225–231**: taxon naming should be checked carefully: "Ascidiaceae," "Molgulide," and "Styledidae" likely need correction/standardization.

**Lines 253–271**: this section is interesting but currently too definitive. The absence of positive temperature correlations does not establish that increases are not temperature-related.

**Lines 272–290**: this is the conceptual core of the paper, but the interpretation needs more explicit acknowledgement that the evidence is indirect.

**Lines 301–307**: well stated overall.

**Lines 308–319**: the practical monitoring implications are promising, but please avoid over-generalizing from a single site/marker/sampling design.

**Lines 320–353**: this section is useful and should perhaps be moved earlier in the Discussion because it provides important context for interpreting the results.

**Lines 354–364**: strong concluding point, this section is persuasive.

**Lines 368–374**: please check "*cells per millilitre*" for zooplankton abundance, this seems unusual and may be an error in units.

**Lines 369–370**: were the two replicate net samples averaged before analysis? Please, state explicitly.

**Lines 379–388**: helpful detail on extraction, but contamination control is not described.

**Lines 389–398**: please state whether PCR negatives, extraction blanks, or field blanks were used and how they were assessed.

**Lines 399–407**: the manual taxonomic curation procedure needs clearer reproducibility criteria.

**Lines 410–417**: please describe the detrending method explicitly and define exactly which chlorophyll/temperature series were used.

**References**

A few references and formatting details should be checked:

**Lines 439–441**: page range appears incorrect ("805-1815"). Should it be 1805?

**Lines 529–530**: page range "188-3201" appears incorrect. Should be 3188?

**Lines 538–539**: "Eco system" should be Ecosystem?

This is a really great study, based on an unusually strong paired time series and addressing an important ecological question. I hope my comments and suggestions improve the manuscript to be published in *Communication Biology*.

Thank you for the opportunity to review this interesting work :)

Best,

Érica Becker.
