# Supplementary material for: eDNA and microscopy reveal traits underlying long-term zooplankton community change
Source: Commun Biol. 2026 Oct 27;9:1409. doi: 10.1038/s42003-026-10901-w (PMC13714307; doi:10.1038/s42003-026-10901-w)
Supplement: Supplementary file 2 [file 42003_2026_10901_MOESM2_ESM.pdf]

Supplementary Table 1: Zooplankton data derived using eDNA can provide enhanced insight into community composition

The numbers of ASVs assigned to each taxonomic group, together with their limits of taxonomic identification (Tax. column; P = Phylum; C = Class, Sc = Subclass, O = Order, Se = Section, Family = F, G = Genus, species = S) are compared to taxonomic data derived from morphological classifications. For some taxonomic groups (highlighted with *), the taxonomist can identify some but not all individuals within the sample; a sub-set is classified as unidentified. For eDNA data, the average relative sequence abundance (%) for each taxonomic group is recorded, alongside the numbers of ASVs assigned to the group. The number of taxa identified by morphology is also shown, alongside average proportional abundance and, where available, biomass (nd = no data available). As no biomass data are available for individual Medusae taxa, this is combined to produce a single value for all Medusae. To facilitate comparisons among the three datasets, the ten most abundant taxa in the eDNA dataset were ranked and highlighted in shades of orange (dark orange = highest rank; light orange = tenth rank), while the ten most abundant taxa based on microscope-derived abundance and biomass were ranked and highlighted in shades of blue (dark blue = highest rank; light blue = tenth rank).

	eDNA data				Microscope Data					
	RSA (%)	Rank	No. ASVs	Tax.	Abund. (%)	Rank	Biomass (%)	Rank	no. taxa	Tax.
ANNELIDA										
Polychaeta	1.4	7	100	F	1		4.1	10	1	P(G)
Sipuncula	0.02		3	F	-	-	-	-	-	-
ARTHROPODA										
Branchiopoda	0.1		3	G	4.2	7	2.6		3	G
Copepoda										
Calanoida	68.3	1	17	G/S	26.6	2	35.9	1	27	S
Canuelloida	0.07		3	G	0.04		nd		1	G
Cyclopoida	11.7	2	29	F/G	29.1	1	7	4	3	G/S
Harpacticoida	0.6	10	8	F/G	1.8	10	1.2		3	G/S
Siphonostomatoida	<0.01		1	F	<0.01		<0.01		1	O
Malacostraca										
Amphipoda	0.01		2	O	4.8	5	0.3		3	So
Cumacea	-		-	-	<0.01		0.03		1	O
Decapoda	0.3		7	Se/F	1.4		5.4	8	?	O/F*
Euphausiidae	-		-	-	0.05		0.6		1	F
Isopoda	0.4		13	O	0.02		0.8		5	O
Thecostraca	0.09		6	O	14.5	3	6.4	5	1	Sc
BRYOZOA	0.03		11	F	0.9		0.18		1	P
CHAETOGNATHA	1.3	8	6	F	1.7		5.6	7	2	G*
CHORDATA										
Appendicularia	3.9	4	7	F	6.2	4	5.8	6	2	F*
Ascidiacea	1.3	8	15	F	<0.01		nd		1	C

Doliolida	0.3		1	F	0.4		0.2		1	O
Salpa	-		-	-	<0.01		nd		1	G
Teleostei	0.08		11	O	0.19		10.4	2	1	C
CNIDARIA										
Anthozoa	0.4		14	O	0.07		0.2		1	C
Anthoathecata	0.1		10	F	0.3		↑		18	G
Leptothecata	0.2		18	F	0.7				10	G
Limnomedusae	0.8	5	1	G	0.6	9	4.6	9	1	G
Narcomedusae	0.4		1	G	0.1				1	G
Trachymedusae	0.04		1	G	<0.01				1	G
Scyphozoa	0.02		6	G	<0.01		↓		2	C*
Siphonophorae	4.1	3	4	F	0.3	8	7.3	3	4	G
CTENOPHORA	1.5	6	5	F	0.13		nd		3	G*
ECHINODERMATA	1.3	8	16	C/O	4.4	6	0.3		4	C
HEMICHORDATA	<0.01		1	C	0.03		0.1		1	P
MOLLUSCA										
Bivalvia	0.8	9	34	O	1.5		0.1		1	P
Cephalopoda	<0.01		3	O	<0.01		nd		-	-
Gastropoda	0.1		31	O/F	0.7		0.08		?	P
Pteropoda	0.07		1	F	0.7		0.08		3	G
Polyplacophora	<0.01		3	C	-		-		-	-
NEMATODA	<0.01		6	C	<0.01		nd		1	C
NEMERTEA	0.1		16	C	0.04		nd		1	P
PHONORIDA	0.1		2	O	0.03		nd		1	P
PLATYHELMINTHES	0.08		4	F	<0.01		nd		1	P
PORIFERA	<0.01		10	C	-		-		-	-

Supplementary Figure 1: Heatmap comparisons of occurrence patterns within the eDNA- and microscope-derived time-series data for Copepoda The monthly average abundance (relative sequence abundance (RSA; red) (%) for eDNA-derived data and no. m-3 (for microscope-derived data; grey) for 2012 to 2022 were calculated. Months are 1 = Jan; 2 = Feb; 3 = Mar and so on. For molecular data, shown are the number of ASVs and sequences within each taxon. X shows significant Spearman correlations between molecular and eDNA data, calculated using month/year averages (n = 125). Y shows Spearman correlation data calculated using annual averages (n = 11). Grey boxes indicate p < 0.05. The terns n.mol (no match in molecular data) and n.mol (no match in microscope data) are used to indicate species or taxonomic groups that were absent from the eDNA data (orange text) or the microscope data (blue text). Nd = no data available.

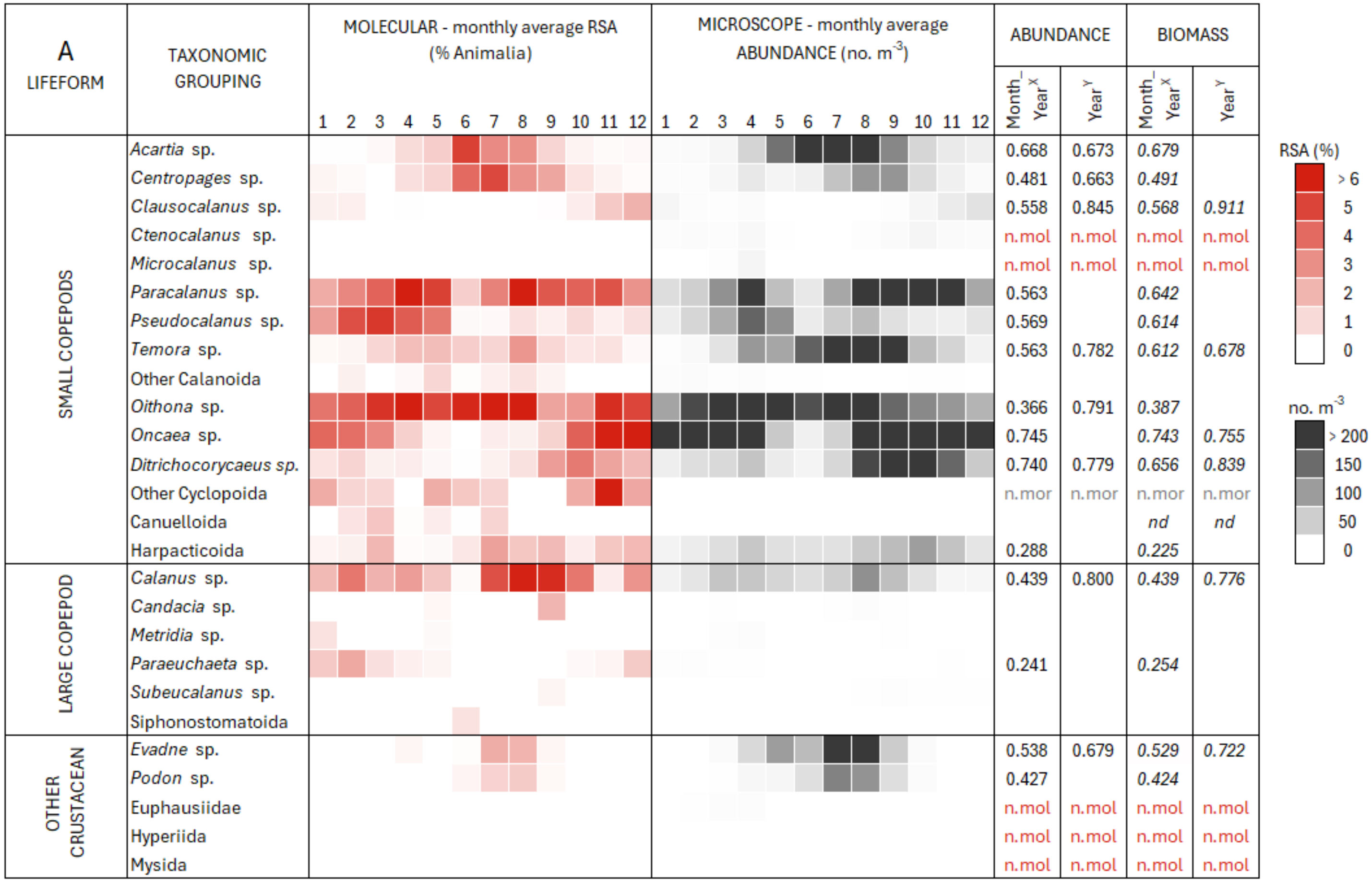

Supplementary Figure 2: Heatmap comparisons of occurrence patterns within the eDNA- and microscope-derived time-series data for zooplankton classified as Gelatinous Predators, Semi-Gelatinous Predators and Other Crustaceans The monthly average abundance (relative sequence abundance (RSA; red) (%) for eDNA-derived data and no. m-3 (for microscope-derived data; grey) for 2012 to 2022 were calculated. Months are 1 = Jan; 2 = Feb; 3 = Mar and so on. For molecular data, shown are the number of ASVs and sequences within each taxon. X shows significant Spearman correlations between molecular and eDNA data, calculated using month/year averages (n = 125). Y shows Spearman correlation data calculated using annual averages (n = 11). Grey boxes indicate $p < 0.05$. The terns n.mol (no match in molecular data) and n.mol (no match in microscope data) are used to indicate species or taxonomic groups that were absent from the eDNA data (orange text) or the microscope data (blue text). Nd = no data available.

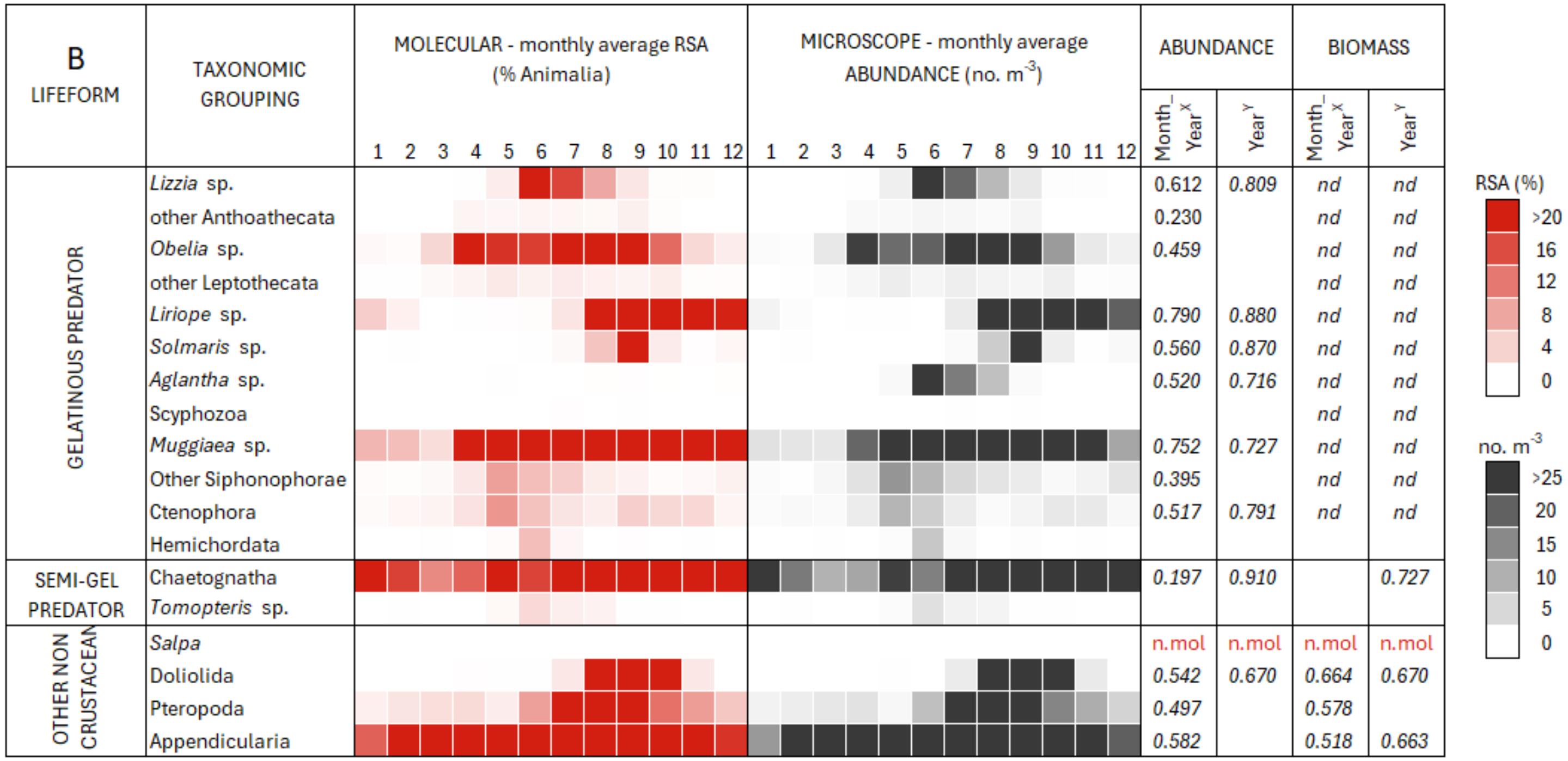

Supplementary Figure 3: Heatmap comparisons of occurrence patterns within the eDNA- and microscope-derived time-series data for zooplankton classified as Meroplankton The monthly average abundance (relative sequence abundance (RSA; red) (%) for eDNA-derived data and no. m-3 (for microscope-derived data; grey) for 2012 to 2022 were calculated. Months are 1 = Jan; 2 = Feb; 3 = Mar and so on. For molecular data, shown are the number of ASVs and sequences within each taxon. X shows significant Spearman correlations between molecular and eDNA data, calculated using month/year averages (n = 125). Y shows Spearman correlation data calculated using annual averages (n = 11). Grey boxes indicate p < 0.05. The terns n.mol (no match in molecular data) and n.mol (no match in microscope data) are used to indicate species or taxonomic groups that were absent from the eDNA data (orange text) or the microscope data (blue text). Nd = no data available.

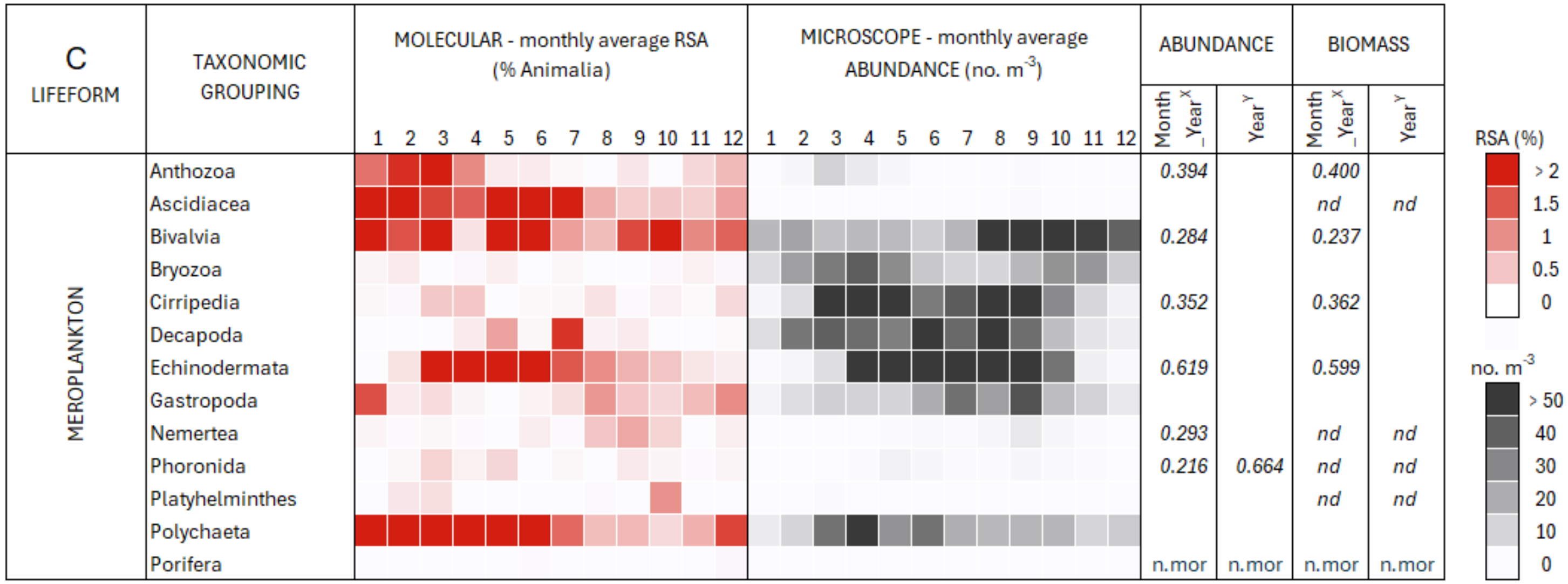

Supplementary Table 2: References used to assign zooplankton feeding groups

**Taxa, organised by feeding trait**	**Reference**
**FINE FILTER FEEDERS**	
Appendicularia	Heneghan, R.F.*et al.* Climate-driven zooplankton shifts cause large-scale declines in food quality for fish. *Nat. Clim. Chang.* **13**, 470–477 https://doi.org/10.1038/s41558-023-01630-7 (2023)
Bivalvia	Hansen, B., Bjornsen, P.K. & Hansen, P.J. The size ratio between planktonic predators and their prey. Limnol. Oceanog. ***39***, 395-403 (1994).
Bryozoa	Devèze, L. Contribution à l'étude de la nutrition des stades larvaires planctoniques. *I. Rec. Trav. Sta. Mar. Endoume, Fasc*. **8**, 49-54 (1953); Atkins, D. The ciliary feeding mechanism of the cyphonautes larva. *J. Mar. biol. Ass. U.K*. 34, 451-466 (1955).
Decapoda	Fileman, E.S., Lindeque, P.K., Harmer, R.A., Halsband, C. & Atkinson, A. Feeding rates and prey selectivity of planktonic decapod larvae in the Western English Channel. *Mar. Biol.* **161**, 2479-2494 (2014).
Doliolida	Fuchs, H.L. & Franks, P.J. Plankton community properties determined by nutrients and size-selective feeding. Mar. Ecol. Prog. Ser, ***413***, 1-15 (2010)
Gastropoda	Hansen, B., Bjornsen, P.K. & Hansen, P.J. The size ratio between planktonic predators and their prey. Limnol. Oceanog. ***39***, 395-403 (1994).
Limacina sp.	Heneghan, R.F.*et al.* Climate-driven zooplankton shifts cause large-scale declines in food quality for fish. *Nat. Clim. Chang.* **13**, 470–477 https://doi.org/10.1038/s41558-023-01630-7 (2023)
Polychaeta	Hansen, B., Bjornsen, P.K. & Hansen, P.J. The size ratio between planktonic predators and their prey. Limnol. Oceanog. ***39***, 395-403 (1994).
Salpa	Madin, L.P. & Kremer, P. Determination of the filter-feeding rates of salps (Tunicata, Thaliacea). *ICES J. Mar. Sci*. 52, 583-95 (1995); Kiørboe, T. How zooplankton feed: Mechanisms, traits and trade-offs. *Biol. Rev*. **86**, 311-339 (2011)
**CARNIVORES/DETRITIVORES**	
Candacia armata	Mauchline J. The biology of calanoid copepods. *Adv. Mar. Biol.* **33** (1988); Ohtsuka, S., Onbé, T. Evidence of selective feeding on larvaceans by the pelagic copepod *Candacia bipinnata* (Calanoida: Candaciidae). *J. Plank. Res*. **11**, 869-872 (1989).
Chaetognatha	Fuchs, H.L. & Franks, P.J. Plankton community properties determined by nutrients and size-selective feeding. Mar. Ecol. Prog. Ser, ***413***, 1-15 (2010)
Ctenophora	Fuchs, H.L. & Franks, P.J. Plankton community properties determined by nutrients and size-selective feeding. Mar. Ecol. Prog. Ser, ***413***, 1-15 (2010)
Ditrichocorycaeus anglicus	Landry, M.R., Lehner-Fournier, J.M. & Fagerness, V.L. Predatory feeding behavior of the marine cyclopoid copepod *Corycaeus anglicus*. Mar. Biol. ***85***, *1*63-169.(1985).
Fish larvae	Fuchs, H.L. & Franks, P.J. Plankton community properties determined by nutrients and size-selective feeding. Mar. Ecol. Prog. Ser, ***413***, 1-15 (2010)

Medusae	Fuchs, H.L. & Franks, P.J. Plankton community properties determined by nutrients and size-selective feeding. Mar. Ecol. Prog. Ser, ***413,*** 1-15 (2010)
Oncaea spp.	Go, Y.B., Oh, B.C. & Terazaki, M. Feeding behavior of the poecilostomatoid copepods Oncaea spp. on chaetognaths. J. Mar. Sys. ***15,*** 475-482.(1988).
Paraeuchaeta hebes	Mauchline J. The biology of calanoid copepods. Adv. Mar. Biol. 33 (1988).
Siphonophorae	Fuchs, H.L. & Franks, P.J. Plankton community properties determined by nutrients and size-selective feeding. Mar. Ecol. Prog. Ser, ***413,*** 1-15 (2010)
Tomopteris helgolandica	Lebour MV. The food of plankton organisms. *J. Mar. biol. Ass. U.K.* **12**, 644-77 (1992).
**SUSPENSION FEEDERS/OMINVORES**	
Acartia (Acartiura) clausi	Fuchs, H.L. & Franks, P.J. Plankton community properties determined by nutrients and size-selective feeding. Mar. Ecol. Prog. Ser, ***413,*** 1-15 (2010)
Calanus helgolandicus	Djeghri, N. *et al*. High prey-predator size ratios and unselective feeding in copepods: a seasonal comparison of five species with contrasting feeding modes. Prog. Oceanog. ***165***, 63-74 (2018).
Centropages typicus	Calbet A., Carlotti F. & Gaudy R. The feeding ecology of the copepod *Centropages typicus* (Kröyer). Prog. Oceanog. 72, 137-150 (2007).
Cirripedia	Fuchs, H.L. & Franks, P.J. Plankton community properties determined by nutrients and size-selective feeding. Mar. Ecol. Prog. Ser, ***413,*** 1-15 (2010)
Clausocalanus spp	Fuchs, H.L. & Franks, P.J. Plankton community properties determined by nutrients and size-selective feeding. Mar. Ecol. Prog. Ser, ***413,*** 1-15 (2010)
Ctenocalanus vanus	Fuchs, H.L. & Franks, P.J. Plankton community properties determined by nutrients and size-selective feeding. Mar. Ecol. Prog. Ser, ***413,*** 1-15 (2010)
Echinodermata	Hansen, B., Bjornsen, P.K. & Hansen, P.J. The size ratio between planktonic predators and their prey. Limnol. Oceanog. ***39,*** 395-403 (1994).
Euterpina acutifrons	Broglio E., Saiz E., Calbet A., Trepat I. & Alcaraz M. Trophic impact and prey selection by crustacean zooplankton on the microbial communities of an oligotrophic coastal area (NW Mediterranean Sea). Aquat. Microb. Ecol. **35,** 65-78 (2004).
Evadne spp.	Fuchs, H.L. & Franks, P.J. Plankton community properties determined by nutrients and size-selective feeding. Mar. Ecol. Prog. Ser, ***413,*** 1-15 (2010)
Oithona spp.	Djeghri, N. *et al*. High prey-predator size ratios and unselective feeding in copepods: a seasonal comparison of five species with contrasting feeding modes. Prog. Oceanog. ***165***, 63-74 (2018).
Paracalanus parvus	Fuchs, H.L. & Franks, P.J. Plankton community properties determined by nutrients and size-selective feeding. Mar. Ecol. Prog. Ser, ***413,*** 1-15 (2010)

Podon spp.	Katechakis, A. & Stibor, H. Feeding selectivities of the marine cladocerans Penilia avirostris, Podon intermedius and Evadne nordmanni. Mar. Biol. ***145***, 529-539 (2004).
Pseudocalanus elongatus	Djeghri, N. *et al*. High prey-predator size ratios and unselective feeding in copepods: a seasonal comparison of five species with contrasting feeding modes. Prog. Oceanog. ***165***, 63-74 (2018).
Temora longicornis	Fuchs, H.L. & Franks, P.J. Plankton community properties determined by nutrients and size-selective feeding. Mar. Ecol. Prog. Ser, ***413***, 1-15 (2010)
