# Supplementary material for: eDNA and microscopy reveal traits underlying long-term zooplankton community change
Source: Commun Biol. 2026 Oct 27;9:1409. doi: 10.1038/s42003-026-10901-w (PMC13714307; doi:10.1038/s42003-026-10901-w)
Supplement: Supplementary file 3 [file 42003_2026_10901_MOESM3_ESM.pdf]

Description of Additional Supplementary Files

File name: Supplementary Data 1

Description: In addition, details of Animalia ASVs identified in this study, including a representative sequence, taxonomic classification together with the ASV table are within Supplementary Data.

File name: Supplementary Data 2

Description: Source Data used to generated Figures 1a, 1b, 2, 3 and 4 can be found in Supplementary Data 2
